# Repurposing ICG enables MR/PA imaging signal amplification and iron depletion for iron-overload disorders

**DOI:** 10.1126/sciadv.abl5862

**Published:** 2021-12-17

**Authors:** Huirong Lin, Yu Zhou, Jiaming Wang, Huimeng Wang, Tianhong Yao, Hu Chen, Huili Zheng, Yang Zhang, En Ren, Lai Jiang, Chengchao Chu, Xiaoyuan Chen, Jingsong Mao, Fudi Wang, Gang Liu

**Affiliations:** 1 State Key Laboratory of Molecular Vaccinology and Molecular Diagnostics and Center for Molecular Imaging and Translational Medicine, School of Public Health, Xiamen University, Xiamen 361102, China.; 2 The Fourth Affiliated Hospital, School of Public Health, Institute of Translational Medicine, Zhejiang University School of Medicine, Hangzhou 310058, China.; 3 Amoy Hopeful Biotechnology Co. Ltd., Xiamen 361027, China.; 4 Departments of Diagnostic Radiology, Chemical and Biomolecular Engineering, and Biomedical Engineering, Yong Loo Lin School of Medicine and Faculty of Engineering, National University of Singapore, Singapore, Singapore.; 5 Department of Radiology, Xiang’an Hospital of Xiamen University, Xiamen 361102, China.; 6 The First Affiliated Hospital, Hengyang Medical School, University of South China, Hengyang 421001, China.

## Abstract

Precise and noninvasive theranostic methods to quantify and deplete focal iron are of crucial importance for iron-overload disorders. Here, we developed an indocyanine green (ICG)–based imaging platform to reveal Fe^3+^ in vitro and in vivo. The high sensitivity and specificity of ICG-Fe interaction facilitated MR images with a marked correlation between *T*_1_ signal intensity ratio (*T*_1_SIR) changes and Fe^3+^ concentration in rodent models and humans. On the basis of these findings, a rational design for coordination-driven self-assembly ICG-Lecithin (ICG/Leci) was proposed to determine Fe^3+^. The enhancement of photoacoustic signal at 890 nm with increasing Fe^3+^ concentration showed an over 600% higher linear slope than that of *T*_1_SIR changes in animal models. ICG/Leci also promoted a 100% increase in iron depletion in the liver compared with deferoxamine. The high MR sensitivity and superior photoacoustic contrast, combined with enhanced iron depletion, demonstrate that ICG/Leci is a promising theranostic agent for simultaneous detection and treatment of iron-overload disorders.

## Teaser

Indocyanine green was repurposed as iron theranostics for simultaneous detection and treatment of iron-overload disorders.

## INTRODUCTION

Iron is an essential element for a variety of metabolic processes in most living systems (*1*). Many common human diseases such as hemochromatosis and chronic hepatitis account for excess iron accumulated in organs (e.g., liver) and cause oxidative damage (*2*, *3*). Methods for the diagnosis of insidious iron-overload disorders often prevent a timely protective intervention leading to progressive and irreversible end-liver injury (*4*–*6*). Magnetic resonance imaging (MRI) is the most commonly used imaging method to diagnose and study liver iron concentration (LIC) in humans. Implementing conventional MRI procedures to detect hepatic iron stores noninvasively leads to precise information on the spatial distribution of iron deposition (*7*–*10*), which is difficult for liver biopsy measurements or other in vitro diagnostic methods (*11*). However, most MR-based iron assessment methods are limited by their inability to distinguish different iron forms, such as labile iron, ferritin, and hemosiderin (*12*–*14*), as well as coexisting fat and massive iron deposition; another limitation is their motion artifacts caused by time-consuming sequences (*15*). These limitations complicate the workflow, particularly their lack of specificity and sensitivity for iron quantification. Therefore, there is an ever-increasing interest in using probe-based signal amplification strategies that generate higher target-to-background contrast for precise detection of LIC.

**Fig. 1.**
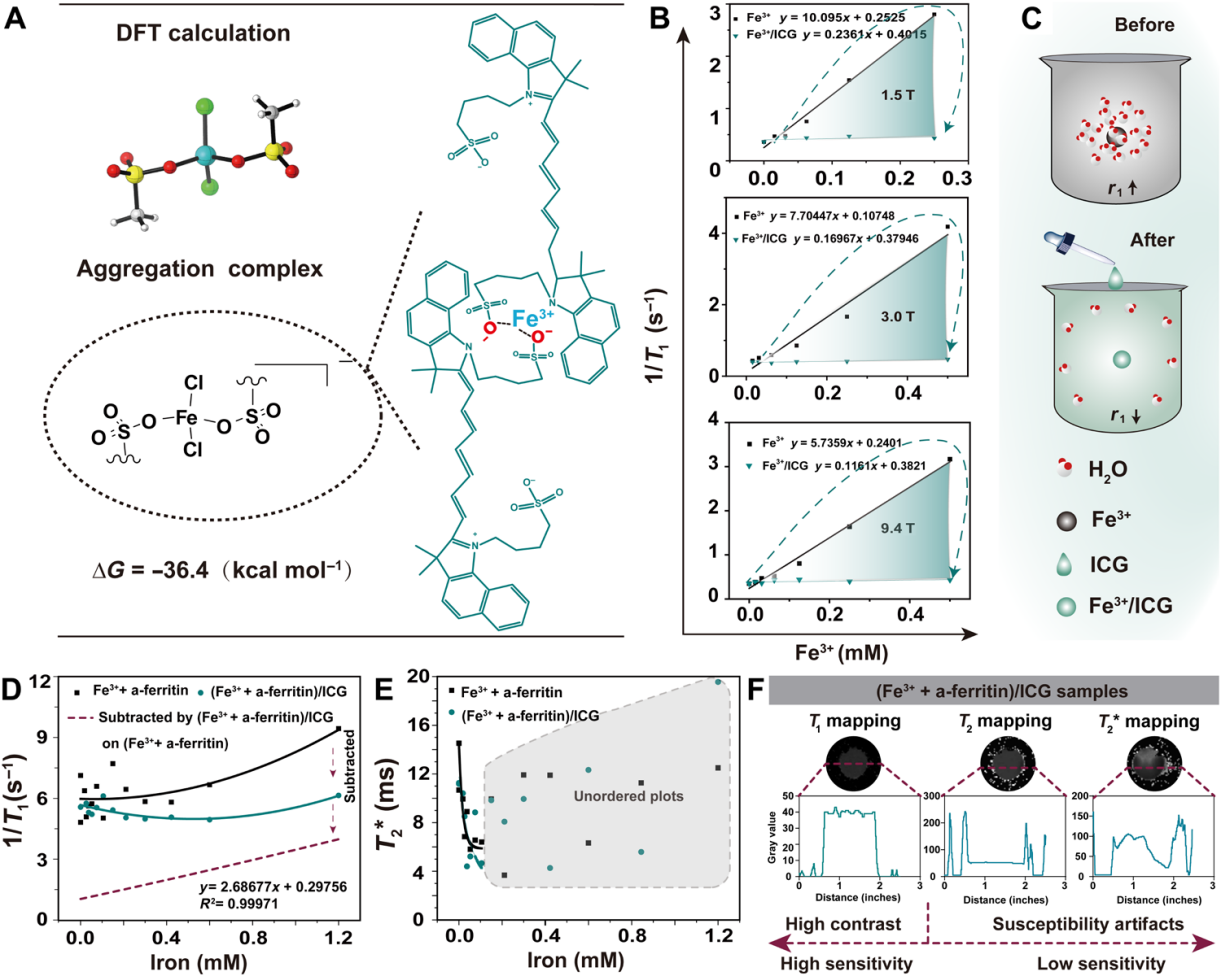
Mechanisms and characterizations of ICG for MRI of Fe^3+^. (**A**) Simplified model of the aggregation complexes between Fe^3+^ and ICG in the DFT calculation (left) and schematic of molecular structure of ICG assembled with Fe^3+^ (right). (**B**) The correlation between *T*_1_ and iron concentration of free Fe^3+^ and Fe^3+^/ICG was measured in 1.5-, 3-, and 9.4-T magnetic fields at pH 4.5. (**C**) Mechanism of water exchange rate detraction around Fe^3+^ as a function of increasing concentration of ICG. (**D**) Correlation between *T*_1_ and iron concentration of Fe^3+^ + a-ferritin (Fe^3+^ + a-ferritin)/ICG was measured in 9.4-T magnetic field at pH 6.8. (**E**) *T*_2_* relaxivity of Fe^3+^ + a-ferritin and (Fe^3+^ + a-ferritin)/ICG. (**F**) Representative *T*_1_ mapping, *T*_2_ mapping, and *T*_2_* mapping images of (Fe^3+^ + a-ferritin)/ICG samples (1 mM), and the image pixels were recorded by ImageJ software.

Clinical drug repositioning and repurposing with well-established pharmacokinetic and biosafety profiles offer a valuable route for identifying probe candidates (*16*, *17*). Indocyanine green (ICG) is approved by the U.S. Food and Drug Administration (FDA) and has been used as a standard to dynamically assess liver function in patients (*18*–*20*). ICG is a popular contrast agent for molecular imaging and can solve the limitations of sensitivity and resolution for concomitant anatomic and physiological information (*21*). Notably, ICG with metal complexing properties in the framework of sulfonic acid (R-HSO₃) and Lewis base (-NH) groups could be used for coordinating with iron ions in the applications of multimodal imaging–guided theranostics (*22*–*24*). Because of recent progress in supramolecular assembly (*25*, *26*), it is strongly desirable to develop ICG-based strategies to change the relaxivity of endogenous iron substrates achieving distinguishable contrast for MRI clinical applications. We propose and validate that Fe^3+^ ions could coordinate hydrophilic regions of ICG molecules and obscure water to access the inner Fe^3+^ ions (Fig. 1A), thus reducing the rate of water exchange and changing the *T*_1_ contrast effect of Fe^3+^ ion, which could potentially address the clinical need for high-performance iron quantification agents in LIC tests.

It is believed that ICG molecules assembled with short- and long-range order can display optical properties distinct from the individually dispersed monomers (*27*–*29*), leading to the versatile ICG/Lecithin (ICG/Leci) system designed here. The polymeric ICG not only serves as an imaging probe to overcome the limitations of sensitivity and resolution that a single imaging technique may have but also acts as a platform for theranostics and LIC treatment featuring precise binding efficiency. This, in turn, could simplify the quantification of imaging and therapeutic outcomes. Our experiments demonstrated the emergence of a unique spectroscopic peak (~890 nm) at the photoacoustic imaging (PAI) signal after ICG/Leci was chelated with Fe^3+^. Thus, combining the high spatial resolution supported by MRI and the rich optical contrast of PAI will sensitively predict the degree of iron overload, with three-dimensional (3D) capabilities offering better treatment planning and assessing therapeutic outcomes. Compared with free ICG and deferoxamine (DFO), one of the world’s most prescribed iron depletion drugs, ICG/Leci could more significantly increase iron excretion and reduce serum ferritin levels in vivo. DFO nephrotoxicity featuring the infiltration of inflammatory cells in the kidneys was found in the DFO group, but the good safety of ICG/Leci was demonstrated here. Overall, this ICG-based system could potentially address the critical need for iron deposition theranostics in the clinic.

## RESULTS

### Characterization of ICG chelation with Fe^3+^, MRI in mice, and pilot clinical study

The interaction energy, calculated by Gaussian09, between ICG molecules is −20.2 kcal mol^−1^ (fig. S1). When ferric chloride was added to ICG, several aggregation modes were investigated with density functional theory (DFT) calculations (fig. S2, A and B, and Fig. 1A). In comparison to ^2^Fe-A0 and ^4^Fe-A0, ^6^Fe-A0 is thermodynamically the most stable complex with the most negative Gibbs free energy change (Δ*G*) at −36.4 kcal mol^−1^ for ICG-Fe^3+^. Thus, the addition of ferric chloride to ICG can promote aggregation. To further confirm the aggregation, the binding affinity of Fe^3+^ for ICG was detected by isothermal titration calorimetry (ITC) with a dissociation constant (*K*_d_) of 0.64 μM. Δ*G* was −35.33 kcal mol^−1^ for the first stage, *K*_d_ was 6.91 μM, and Δ*G* was −29.44 kcal mol^−1^ for the second stage (fig. S3A). We also measured *K*_d_ with 6520.49 μM and Δ*G* at −12.47 kcal mol^−1^ for Fe^3+^-DFO (fig. S3B), which indicated that ICG has a much higher affinity than DFO (a clinical chelating agent used to remove excess iron) to Fe^3+^ under this condition.

In the presence of ICG, there was a significant decrease in the *r*_1_ value of Fe^3+^, which was approximately 50-fold lower than that of free Fe^3+^ under the MRI scanner (Fig. 1B). This phenomenon might be ascribed to the hydrophilic regions of ICG bound to Fe^3+^ with water obscured to access the inner Fe ions, which reduced the rate of water exchange over free Fe^3+^ (Fig. 1C). Therefore, a longer spin-lattice relaxation time and a lower *r*_1_ are consistent with the reported theories (*30*–*32*). However, the *r*_1_ value in phantoms with hemosiderin-like aggregated ferritin (a-ferritin) (fig. S4A) was independent of the added ICG (fig. S4B). The *r*_1_ value of Fe^3+^/a-ferritin hybrid decreased with a significant value of 2.7 mM^−1^ subtracted from (Fe^3+^ + a-ferritin) to (Fe^3+^ + a-ferritin)/ICG (Fig. 1D). The *r*_1_ value of free Fe^3+^ was close to 2.9 mM^−1^ in the same solution (fig. S4C). In contrast, normative *T*_2_* values of (Fe^3+^ + a-ferritin) and (Fe^3+^ + a-ferritin)/ICG showed no significant difference (Fig. 1E). Furthermore, *T*_2_* values were not significantly correlated with changes in iron concentration (>0.1 mM) probably due to the strong susceptibility artifact under high field strength (Fig. 1F). This indicated that ICG can be repurposed as a *T*_1_ MRI probe activated by Fe^3+^ with strong image contrast and detect Fe^3+^ in a quick and noninvasive way.

**Fig. 2.**
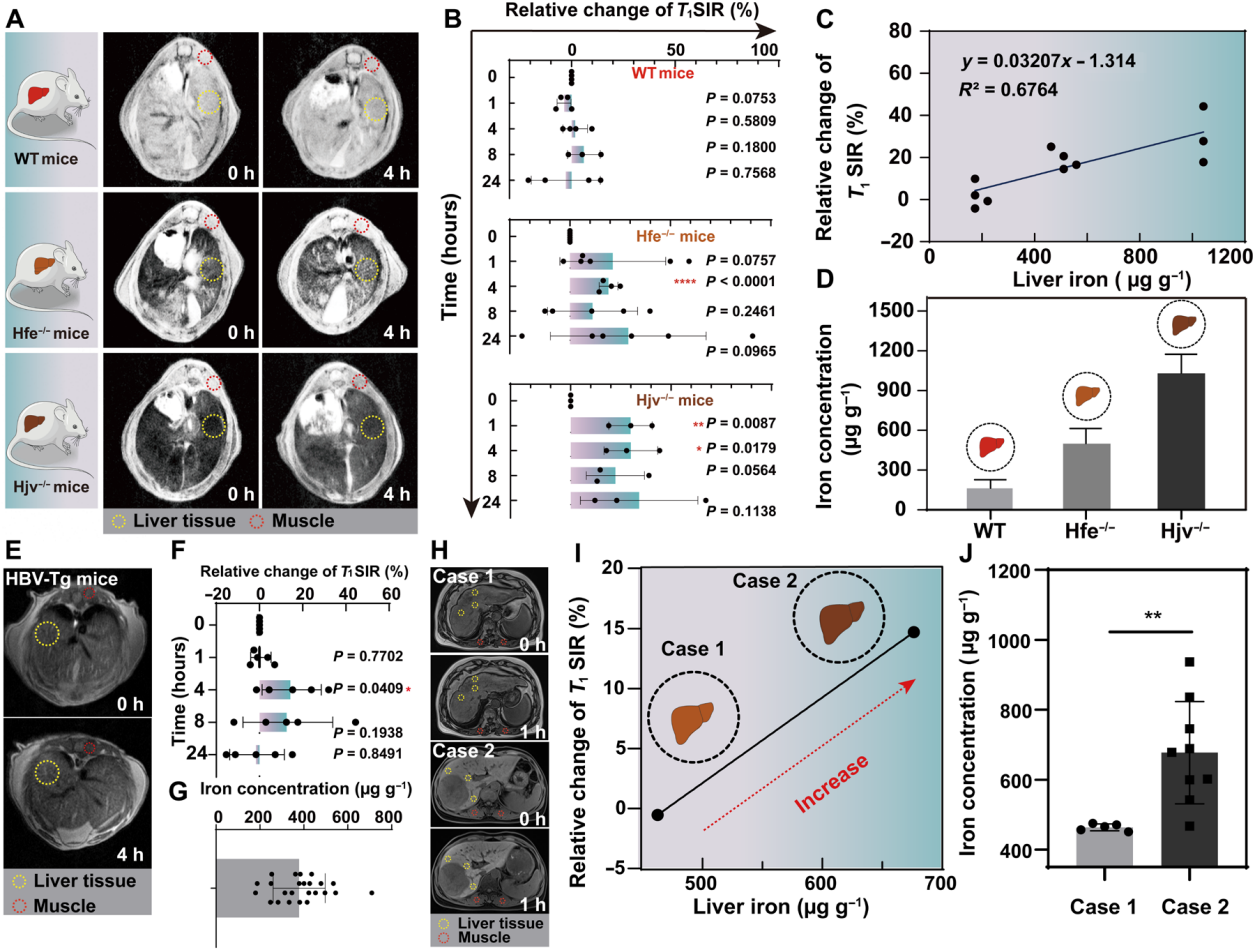
MRI of iron fluxes by ICG in vivo. (**A**) Representative liver MRI images of WT, Hfe^−/−^, and Hjv^−/−^ mice at 0 and 4 hours after injection of ICG (ICG dose, 2.5 mg kg^−1^). (**B**) Calculated relative changes of *T*_1_SIR in livers of WT, Hfe^−/−^, and Hjv^−/−^ mice at 0, 1, 4, 8, and 24 hours after injection of ICG (ICG dose, 2.5 mg kg^−1^). Relative change of *T*_1_SIR = 100 × [(*T*_1_SI_M_/*T*_1_SI_L_)^0h^ **−** (*T*_1_SI_M_ / *T*_1_SI_L_)^nh^]/(*T*_1_SI_M_ / *T*_1_SI_L_)^0h^. *T*_1_SI_M_, *T*_1_SI in muscle; *T*_1_SI_L_, *T*_1_SI in the liver. (**C**) MRI response of ICG to the reactive Fe^3+^ of various concentrations in WT, Hfe^−/−^, and Hjv^−/−^ mice. (**D**) Iron concentration in livers of different kinds of mice. (**E**) Representative liver MRI images of HBV-Tg mice at 0 and 4 hours after injection of ICG (ICG dose, 2.5 mg kg^−1^). (**F**) Calculated relative changes of *T*_1_SIR in livers of HBV-Tg mice at 0, 1, 4, 8, and 24 hours after injection of ICG (ICG dose, 2.5 mg kg^−1^). Relative change of *T*_1_SIR = 100 × [(*T*_1_SI_M_/*T*_1_SI_L_)^0h^ **−** (*T*_1_SI_M_/*T*_1_SI_L_)^nh^]/(*T*_1_SI_M_/*T*_1_SI_L_)^0h^. (**G**) Iron concentration in the livers of HBV-Tg mice. (**H**) Representative liver MRI images of patients with chronic viral hepatitis–related hepatocellular carcinoma. LIC was tested with MRI at 0 and 1 hour after receiving intravenous ICG (0.5 mg kg^−1^). (**I**) The ROIs were placed in the liver tissues and muscles, and the relative change of *T*_1_SIR = 100 × [(*T*_1_SI_M_/*T*_1_SI_L_)^0h^ **−** (*T*_1_SI_M_/*T*_1_SI_L_)^nh^]/(*T*_1_SI_M_/*T*_1_SI_L_)^0h^ was calculated. (**J**) Iron concentration of the liver tissues was detected by ICP-MS. Mean ± SD; *n* = 5 to 8 per group; *P < 0.05, **P < 0.01, ****P < 0.0001 by unpaired *t* test (two-tailed).

The ability to use ICG for MRI and iron detection in vivo was first demonstrated in mouse liver tissues with Fe^3+^ or ICG-Fe^3+^ injection (fig. S5, A and B). Then, two different iron-overload phenotype knockout mice, Hfe^−/−^ mice and Hjv^−/−^ mice (*33*), were used to acquire the information of signal change corresponding to different iron deposited levels in livers and relevant to in vivo iron-overload disorders. Figure 2A shows representative *T*_1_ signal images from the wild-type (WT), Hfe^−/−^, and Hjv^−/−^ mice at 4 hours after intravenous injection of ICG. Figure 2B shows the relative change of *T*_1_SI_M_ (*T*_1_ signal intensity in muscle)/*T*_1_SI_L_ (*T*_1_ signal intensity in liver) for mice treated with ICG. At 0-, 1-, and 4-hour time points after treatment with ICG, the signal from Fe^3+^ in the liver of WT mice showed only subtle changes from 0, −3.61, to 1.74%. However, the signal changes in the Hfe^−/−^ and Hjv^−/−^ mice showed more apparent changes of 19.19 and 29.97% at 4 hours, respectively. Linear regression analysis showed that mice treated with ICG had an iron concentration–dependent increase (WT mice, 173.80 ± 62.81 μg of iron per gram of liver weight; Hfe^−/−^ mice, 510.90 ± 64.86 μg of iron per gram of liver weight; Hjv^−/−^ mice, 1042.00 ± 63.25 μg of iron per gram of liver weight) with a regression line of *y* = 0.03207*x* − 1.314 in *T*_1_SI_M_/*T*_1_SI_L_ change at 4 hours (Fig. 2, C and D).

We also examined the *T*_1_ signal response in hepatitis B virus transgenic (HBV-Tg) mice with hepatic iron overload (Fig. 2E) (*34*, *35*). After injection with ICG (2.5 mg kg^−1^), *T*_1_SI_M_/*T*_1_SI_L_ of the liver tissues in HBV-Tg mice showed a 14.91% change of the relative *T*_1_SI at 4 hours versus 0 hours (Fig. 2F). Moreover, LIC of 395.04 ± 79.28 μg g^−1^ tested by atomic absorption spectrometry was in good agreement with that from the MRI measurement (Fig. 2G). Note that mild-to-moderate hepatic iron overload is common in patients with chronic viral hepatitis, and iron overload is related to the severity of liver disease, but liver biopsy is usually not performed because of ethical restrictions. Thus, the patients with chronic viral hepatitis–related hepatocellular carcinoma were tested in a clinical MRI system after being administered with ICG (0.5 mg kg^−1^) before surgical removal of the tumor (Fig. 2H). Relative to −3.20% change of *T*_1_SI of liver tissues in the health volunteer (fig. S6, A and B), the *T*_1_ signal response in case 1 showed a −0.5% change at 1 hour versus 0 hour, while there was a 14.71% change in case 2 (Fig. 2I), and the corresponding LICs were 463.89 ± 10.03 μg g^−1^(mild positive Prussian blue staining) and 677.54 ± 146.31 μg g^−1^ (moderate positive Prussian blue staining), respectively (Fig. 2J and fig. S7). These results are consistent with the findings of liver response in mice, demonstrating that ICG can be repurposed as a potential contrast agent for quantitative MRI in iron-overload conditions.

### Photophysical property changes of ICG

**Fig. 3.**
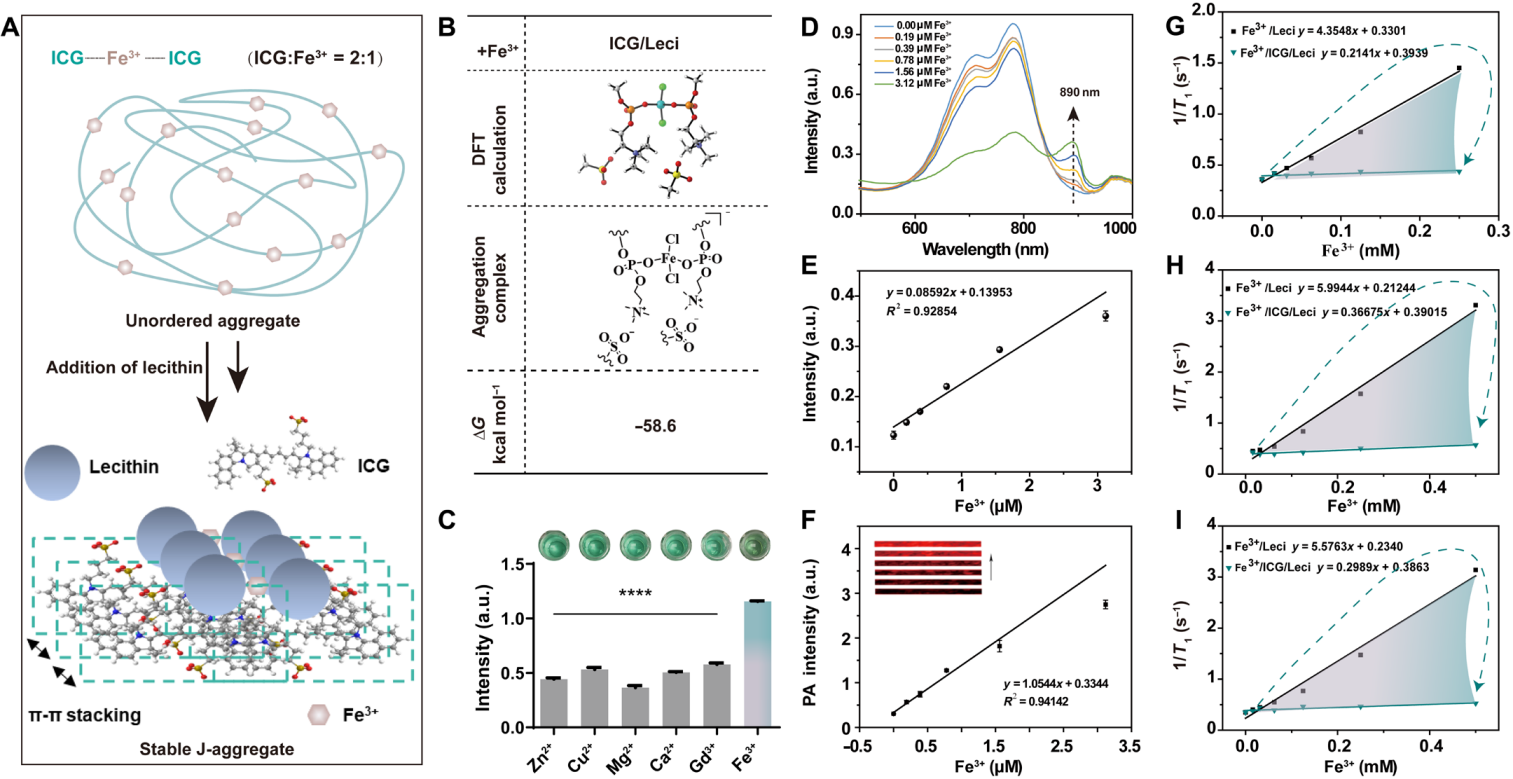
Design, mechanisms, and characterizations of ICG/Leci for Fe^3+^ imaging. (**A**) Schematic of J-aggregation mechanisms of ICG during Fe^3+/^ICG/Leci formation, mediated by deposition of Fe^3+^ onto lipid bilayer. (**B**) Simplified models of the aggregation complexes between Fe^3+^ and ICG/Leci in the DFT calculation. (**C**) Selectivity of the sensor was tested by the peak of the absorbance for several metals added at 10 μM to ICG (40 μM)/Leci (80 μM). (**D**) UV-vis absorption spectra of ICG (100 μM)/Leci (200 μM) upon incubation with different concentrations of Fe^3+^ (0 to 3.12 μM) at pH 4.5. (**E**) Plots of absorbance at 890 nm versus biomarker concentration. (**F**) Photoacoustic response of the probes at 890 nm to Fe^3+^ (0 to 3.12 μM) at pH 4.5. *r*_1_ of Fe^3+^/Leci and Fe^3+^/ICG/Leci was measured in 1.5-T (**G**), 3-T (**H**), and 9.4-T (**I**) magnetic fields at pH 4.5. Mean ± SD; *n* = 4 per group; ****P < 0.0001 by unpaired *t* test (two-tailed). a.u., arbitrary units.

Metal chelation can promote ordered aggregation and further influence the optical properties of ICG. Photophysical characterization of ICG in the presence of different Fe^3+^ concentrations exhibited an increasing peak at 875 nm over time (fig. S8, A to J). However, this process is time-consuming and requires a high Fe^3+^ concentration (>3 μM) (fig. S8J). To further promote the aggregation and surmount monomerization challenges from circulating albumin in vivo, Leci was introduced to control the orientation of ICG molecules and thus contribute to the formation of J-aggregation (Fig. 3A). The interaction energy was lowest at −31.2 kcal mol^−1^ after Leci was added into ICG (fig. S9), which was significantly higher than when ferric chloride was added to ICG/Leci (Fig. 3B and fig. S10). The Leci addition could further facilitate the formation of J-aggregates, and the amount of Leci reached saturation at two equivalents (Leci:ICG = 2:1), which is consistent with the experimental results (fig. S11A). We then measured *K*_d_ with 34.49 μM after Fe^3+^ was dropped into ICG/Leci (fig. S11B); high selectivity for Fe^3+^ over Zn^2+^, Cu^2+^, Mg^2+^, Ca^2+^, and Gd^3+^ was demonstrated (Fig. 3C).

Notably, in the presence of Fe^3+^, ICG/Leci exhibited a strong decreasing absorption band from 550 to 800 nm and a marked red shift to 890 nm at pH 4.5 (Fig. 3, D and E). This has lower iron concentration detection limits than at pH 7.4 (fig. S12, A and B) because ICG-available iron is mainly in lysosomes featuring a lower pH than the surrounding cytoplasm (*36*, *37*). Moreover, the changes in absorption ratios, especially under the lower concentration of Fe^3+^, were threefold quicker at pH 4.5 versus pH 7.4 (fig. S12, C and D). However, no noticeable signals at 890 nm were seen in the control groups without adding Fe^3+^ as shown in fig. S13. Consequently, the PA intensity at 890 nm gradually increased upon the addition of Fe^3**+**^ (Fig. 3F and fig. S14A) and a marked fluorescence decline at 672-nm excitation wavelength upon incubation with Fe^3+^ (fig. S14B). Fe^3+^/ICG/Leci retained low fluorescence intensity, which might be primarily due to solid π-π stacking between ICG and the inner Leci shell mediated by Fe^3+^. This interaction contributes to the maximum PA intensity at 890 nm. In the presence of ICG/Leci, we also observed a significant decrease in the value of *r*_1_ of Fe^3+^ (Fig. 3, G to I). These results demonstrated that ICG/Leci could be applied as a multimodal probe for MRI, PAI, and fluorescence imaging to detect and image the corresponding Fe^3+^ noninvasively.

### Cytotoxicity and Fe^3+^ iron imaging of ICG/Leci in vitro

**Fig. 4.**
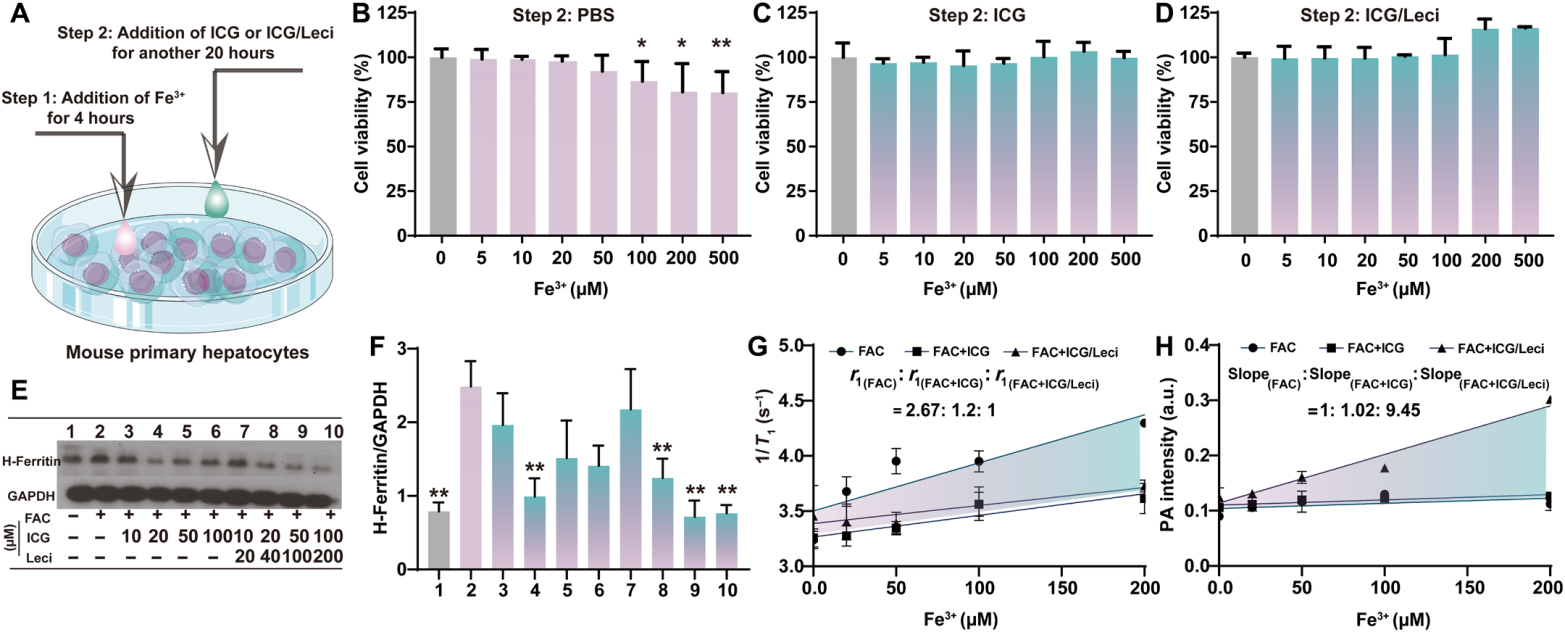
In vitro toxicity, cell signaling regulation, and Fe^3+^ imaging. (**A**) Design of cell experiments. The viability of mouse primary hepatocytes was assessed by MTT analysis after adding Fe^3+^ followed by treatment with PBS (**B**), free ICG (**C**), and ICG/Leci (**D**). The Fe^3+^/ICG/Leci shows no obvious cytotoxicity on mouse primary hepatocytes. (**E**) Western blots were carried out for protein detection, and the amount of protein was quantified, normalized to the amount of GAPDH. (**F**) The addition of ICG/Leci significantly decreases the H-ferritin level with increasing concentration. (**G**) *T*_1_SI of mouse primary hepatocytes following treatment with free Fe^3+^, Fe^3+^/ICG, and Fe^3+^/ICG/Leci was measured in a 9.4-T MRI system. (**H**) Photoacoustic response of mouse primary hepatocyte cells following treatment with free Fe^3+^, Fe^3+^/ICG, and Fe^3+^/ICG/Leci. Mean ± SD; *n* = 4 per group; *P < 0.05, **P < 0.01 by unpaired *t* test (two-tailed).

The in vitro cytotoxicity study with mouse primary hepatocytes was tested by MTT (4-nitrophenyl chloroformate 3-(4,5-dimethylthiazol-2-yl)-2,5-diphenyltetrazolium bromide) and (Cell Counting Kit-8) (CCK8) assays. Fe^3+^ reduced the cell viability as the concentration increased (Fig. 4B and fig. S15B), but there was a treatment effect following the addition of ICG or ICG/Leci (Fig. 4, C and D). As shown in Fig. 4 (E and F) and fig. S15 (C to E), the addition of ICG, especially ICG/Leci, significantly decreases the H-ferritin, ferroportin, and transferrin receptors 1(TfR1) levels with increasing concentration. These results suggest that ICG or ICG/Leci ICG/Leci could rapidly chelate Fe^3+^ iron and then protect cells from the oxidative stress damage caused by iron.

We next investigated the cellular iron chelation imaging with fluorescence, MRI, and PAI. As expected, efficient uptake of probes in cells was observed by the bright red fluorescence images present in fig. S16 (A and B). However, there was a significant reduction of fluorescence intensity in the ICG/Leci group when cells were preincubated with ferric ammonium citrate (FAC) (fig. S16, C and D). In contrast to the free FAC group, few *T*_1_SI changes were observed when treated with different concentrations of ICG or ICG/Leci (Fig. 4G). Noticeable PA signals were observed in ICG/Leci-treated cell groups (Fig. 4H). The *T*_1_SI and PA signals increased with increasing ICG or ICG/Leci concentration. These results further suggest the intracellular iron chelation by ICG/Leci and the possibility of MRI/PA image-guided iron quantitation and depletion in vivo.

### In vivo biodistribution and MRI/PAI of ICG/Leci

**Fig. 5.**
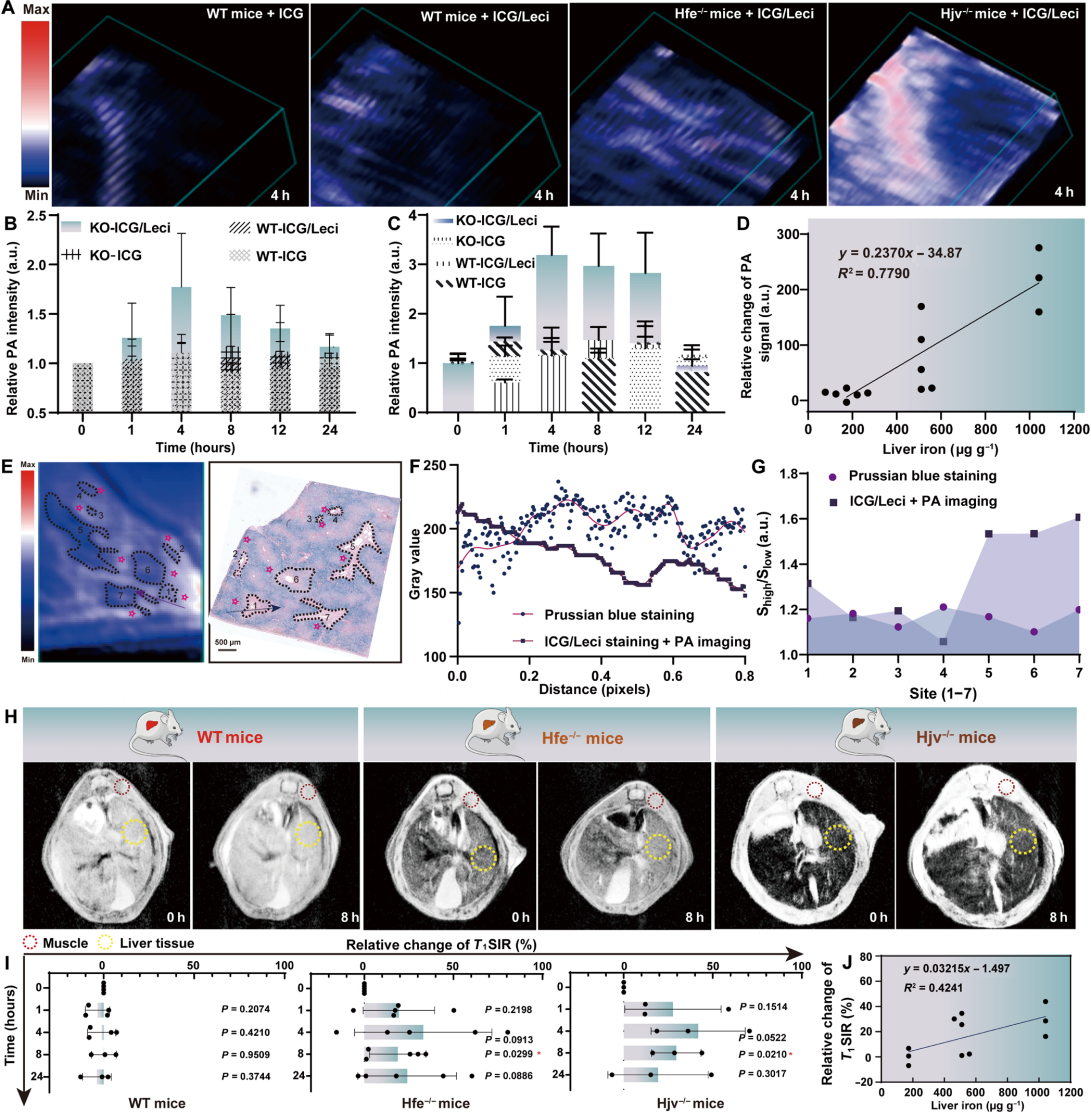
PAI and MRI of iron fluxes by ICG/Leci in livers. (**A**) Representative 3D photoacoustic images of mice models before and after intravenous injection of free ICG and ICG/Leci (ICG dose, 2.5 mg kg^−1^). Quantitative PA intensity at 890 nm in livers of WT, Hfe^−/−^ (**B**), and Hjv^−/−^ mice (**C**) at 0, 1, 4, 8, 12, and 24 hours after injection of ICG and ICG/Leci. KO, knockout. (**D**) Photoacoustic response of ICG/Leci to the reactive Fe^3+^ in mice. (**E**) ICG/Leci staining following PAI (left) and Prussian blue staining (right) of the liver of human with iron overload. The gray value was changing tendency (arrows) (**F**) and relative signal in the ROI (**G**). (**H**) Representative MRI images of MRI signal in livers of mice at 0 and 8 hours after injection of ICG (ICG dose, 2.5 mg kg^−1^). (**I**) Calculative relative changes of *T*_1_SIR in livers of mice at 0, 1, 4, 8, and 24 hours after injection of ICG (ICG dose, 2.5 mg kg^−1^). Relative change of *T*_1_SIR = 100 × [(*T*_1_SI_M_/*T*_1_SI_L_)^0h^ **−** (*T*_1_SI_M_/*T*_1_SI_L_)^nh^]/(*T*_1_SI_M_/*T*_1_SI_L_)^0h^. (**J**) MRI response of ICG/Leci to the reactive Fe^3+^ in vivo. Mean ± SD; *n* = 5 per group; *P < 0.05 by unpaired *t* test (two-tailed).

Before MRI/PAI, the biodistribution of ICG and ICG/Leci in the livers of WT and Hfe^−/−^ mice was recorded by fluorescence at 672 nm. Figure S17 (A and B) shows that the liver fluorescence intensity in the ICG/Leci group was much higher than that of the ICG group in the WT mice, but it decreased more quickly in the Hfe^−/−^ mice with time. It is believed that this is due to the increasing π-π stacking among ICG molecules under activation of excess Fe^3+^. Figure S18 (A and B) shows the co-registered ultrasound + PA images of Hjv^−/−^ mice from 0-, 1-, 4-, 8-, 12-, to 24-hour time points after intravenous injection with ICG and ICG/Leci. The PA signal at 890 nm gradually increased. After ICG injection, little PA signal change could be observed. Encouraged by that, more experiments were conducted in WT and Hfe^−/−^ mice with a moderate iron concentration in the liver. The cross-sectional 3D images were reconstructed from the PA signals at 4 hours as shown in Fig. 5A. The results showed the high PA contrast from iron pools adjacent to hepatic vasculatures in Hfe^−/−^ and Hjv^−/−^ mice, which is consistent with the distribution of iron surrounding the vessels among the liver parenchyma as seen in Prussian blue images (fig. S18, C and D). The optical contrast of vessels in the 3D images remained clear in WT mice originating from Hb and HbO_2_. The PAI signal intensity at 890 nm in Hfe^−/−^ and Hjv^−/−^ mice increased with time (Fig. 5, B and C). It was approximately 75 and 225% enhancement compared with control mice at 4 hours, which was probably due to the in situ generation of strong π-π stacking between ICG during Fe^3+^/ICG/Leci. The photoacoustic response of ICG/Leci to corresponding excess reactive Fe^3+^ in WT, Hfe^−/−^, and Hjv^−/−^ mice showed a regression line of *y* = 0.2370*x* − 34.87 (Fig. 5D), with a more than 600% higher slope value than that in MRI (*y* = 0.03207*x* − 1.314) (Fig. 2C). In addition, 2 weeks after the mice were treated with DFO, the PA signal in Hfe^−/−^ mice remained steady at 4 hours but showed a 50% increase in the DFO group (fig. S19, A to C).

To verify the potential clinical application of ICG/Leci as a PAI contrast agent for iron deposited in the liver, we next screened tissue specimens from the iron-overload patients confirmed by liver biopsy measurements. No PA signal was observed before ICG/Leci staining (fig. S20A). On the contrary, the PA signal was positive when the iron-deposited section was stained (Fig. 5E, left). The shapes of the positive area generally matched those of the positive area of the corresponding Prussian blue staining (Fig. 5E, right), but they did not match when stained with free ICG (fig. S20B). The PAI signal and Prussian blue density along the arrows were analyzed with ImageJ, and there was an inverse correlation between the two methods (Fig. 5F). The relative PAI signal intensity between negative and positive areas was the same as or even better than that in the Prussian blue staining section (Fig. 5G), demonstrating that PAI after ICG/Leci staining could discriminate the iron-deposited area from the normal area in clinical imaging.

The ability of ICG/Leci to detect excess iron via MRI was tested in WT, Hfe^−/−^, and Hjv^/−^ mice. Figure 5H shows MRI images via *T*_1_ signal from the WT, Hfe^−/−^, and Hjv^−/−^mice at 0 and 8 hours after intravenous injection of ICG/Leci. The relative changes in *T*_1_SI_M_/*T*_1_SI_L_ signals were 0.2, 18.73, and 29.54% for WT, Hfe^−/−^, and Hjv^−/−^, respectively (Fig. 5I). Next, mice treated with ICG/Leci showed an iron concentration–dependent increase in *T*_1_SI_M_/*T*_1_SI_L_ signal change with a regression line of *y* = 0.03215*x* − 1.497 (Fig. 5J). This is as expected and was consistent with an ICG-dependent response to intra-liver iron levels.

### Iron excretion and elimination studies in vivo

**Fig. 6.**
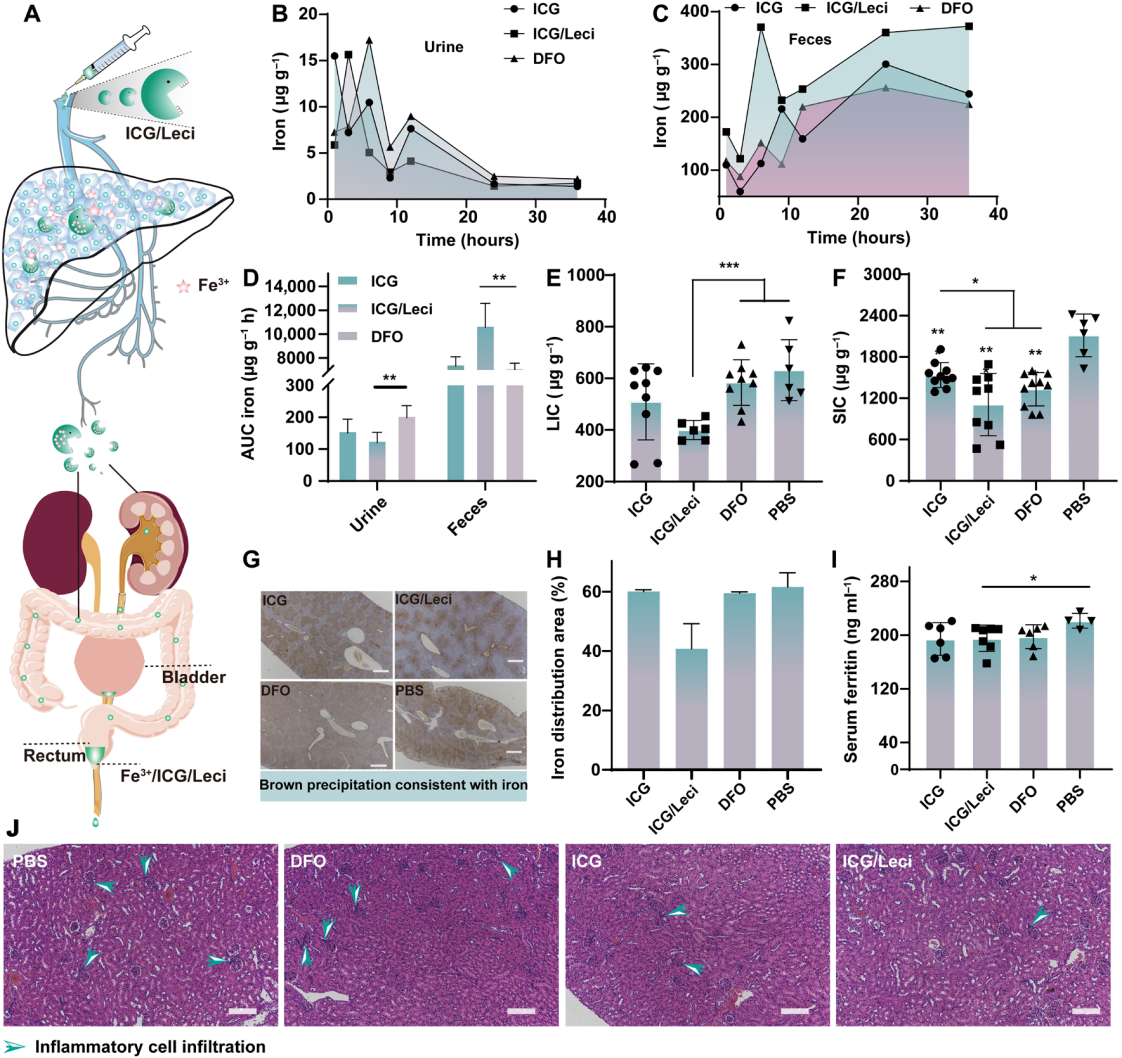
Iron depletion efficacy and nephrotoxicity in vivo. (**A**) Iron chelators of ICG/Leci will efficiently capture hepatic iron after they are intravenously injected into Hfe^−/−^ mice and cleared via urine and feces. Concentration of iron in the urine (**B**) and feces (**C**) of mice at different time points and total iron during 36 hours (**D**). Liver (**E**) and spleen (**F**) iron concentrations were measured in mice following various treatments. (**G**) Enhanced Prussian blue staining: representative liver sections of PBS, DFO, ICG, and ICG/Leci-treated mice. Scale bar, 200 μm, 4×. (**H**) Iron distribution area counted by ImageJ software. (**I**) Serum ferritin level was measured by mouse ferritin ELISA assay of different groups of mice. (**J**) H&E-stained sections of kidney in mice following treatment. Green arrowheads show the infiltration of inflammatory cells. Scale bar, 100 μm. Mean ± SD; *n* = 5 per group; *P < 0.05, **P < 0.01, ***P < 0.001 by unpaired *t* test (two-tailed).

We tested the iron mobilization efficiency in Hfe^−/−^ mouse models by determining urine and feces iron excretion after an equivalent single dose of 2.5 mg kg^−1^ by intravenous injection (Fig. 6, B to D). ICG is known to be excreted in bile in unconjugated form, but DFO is metabolized principally by plasma enzymes. In contrast to DFO, the urine iron content in ICG or ICG/Leci group showed a slight decrease, but the increased excretion of iron conjugation via feces was noted. There was a 1.5-fold higher fecal iron content in mice receiving ICG/Leci, probably leading to minor renal injury, which is one of the most severe and frequent adverse effects of DFO treatment.

Next, we verified the iron mobilization efficiency from the liver and spleen, where iron overloading is predominant based on inductively coupled plasma mass spectrometry (ICP-MS), Western blot, and immunohistochemistry. The iron content is shown in Fig. 6 (E and F). In contrast to placebo [phosphate-buffered saline (PBS)], ICG and DFO were similar and effective at iron depletion from the liver and spleen. As expected, there was the most significant improvement in mobilized iron after ICG/Leci injection, and the iron depletion content was increased by 100% in both the liver and the spleen. The iron level in liver sections was confirmed by DAB (3,3′-diaminobenzidine)–enhanced Prussian blue staining (Fig. 6, G and H). The area of the brown spots was reduced significantly after treatment with ICG/Leci. The data are consistent with the trend in iron content. We further quantified serum ferritin levels by enzyme-linked immunosorbent assay (ELISA; Fig. 6I) and found that serum ferritin concentrations decreased. These results are encouraging and suggest that ICG-based drugs can be iron chelator candidates for efficiently removing excess iron.

### Toxicity in vivo

The in vivo toxicity of ICG/Leci was also characterized by histochemical and blood biochemical analyses. Both the Fenton reaction caused by deposited iron and administration of DFO would lead to renal injury. This helped ensure inflammatory cell infiltration (*38*, *39*). The hematoxylin and eosin (H&E) staining of kidney sections from PBS or DFO-treated mice demonstrated significant inflammatory infiltrate (Fig. 6J). Conversely, kidney sections of ICG-treated mice, especially ICG/Leci-treated mice, showed reduced infiltration. Moreover, neither abnormality nor lesions showed a noticeable difference in other major organs (for example, heart, liver, spleen, and lung) (fig. S21). There were no significant changes of biochemical indexes (cholinesterase, total bile acid, alkaline phosphatase, aspartate aminotransferase, and alanine aminotransferase) noted in the ICG or ICG/Leci-treated group after 2 weeks (fig. S22). These studies suggest that ICG or ICG/Leci can be used as a biocompatible theranostic platform in clinical practice.

## DISCUSSION

One major challenge of MRI is its relatively low sensitivity for the detection of biomarkers with critical cellular/molecular-level information, although applying MRI to estimate LICs has been studied for nearly 40 years. Amplification strategies with endogenous MR contrast (e.g., augmented relaxivity of magnetic substrates–iron) are vital for developing sufficiently sensitive probes for LIC clinical detection. Repurposing approved drugs for LIC detection is a critical resource for potential probes. The initial goal of this study is to determine the ferric ion chelation capability of ICG in situ for signal amplification to decrease the detection limit in LIC mouse models and human subjects; here, high-performance MR measurements could be calibrated via atomic absorption spectrometry and biopsy. This strategy fits with the mechanism of water exchange restrictions around Fe ions influenced via the hydrophobic group of ICG, which leads to a shorter spin-lattice relaxation time and a lower *r*_1_ value (*30*–*32*).

ICG can precisely and accurately identify only one type of iron deposition (i.e., Fe^3+^) in a short time. This indicates a good linear relation between *T*_1_SIR (*T*_1_ signal intensity ratio) and LIC. However, the different types of iron influence *R*_2_ and *R*_2_* relaxometry (conventional standard procedures for iron quantification) in different ways that result in an inaccurate reflection of LIC (*40*), especially the significant susceptibility artifact under high field strengths (*15*, *41*). Even the Carr-Purcell-Meiboom-Gill sequence that could separately estimate ferritin or hemosiderin is limited by long acquisition times and complex calibration (*14*, *42*). ICG is a *T*_1_ MRI active probe and has overcome these challenges by introducing a higher accuracy and simple workflow for noninvasive iron quantitative analysis. In addition, the MRI sequence required for this approach, without complicated parameters and equipment requirements, is accessible in clinical MR settings.

Favorable probes for multimodality imaging harness capabilities to obtain concomitant anatomic and physiological information with high sensitivity and specificity. We therefore proposed and validated that ICG/Leci complexes show unique spectroscopic change (i.e., 890 nm) and produce a detectable PA signal after chelating Fe^3+^. This is likely due to the strong π-π stacking between ICG inner Leci shell mediated by Fe^3+^ (*43*–*45*). MRI provides high spatial resolution for molecular imaging, while PAI has stronger contrast over other optical imaging techniques (*46*). Thus, the co-registration with MR images provides anatomic information for localizing the functional data generated by PAI. As expected, the regression line of the optoacoustic response of ICG/Leci to the corresponding excess Fe^3+^ iron in mice showed an over 600% higher slope value than that in MRI, indicating sensitivity complementation for iron quantification.

To the best of our knowledge, no previous work has introduced a probe for simultaneous iron quantification and depletion in vivo. The ICG/Leci formulas with dual-modality imaging can not only accurately diagnose LIC for better treatment planning but also simultaneously exhibit much higher iron depletion efficacy than DFO following image-guided assessment of therapeutic outcomes. Our results further suggest that polymeric ICG could more significantly increase iron excretion and reduce kidney failure in mice than most extensively used DFO. Notably, DFO or other FDA-approved iron chelators face unfavorable pharmacokinetics and significant toxicity, although they are macromolecules with a long circulation half-life (*39*, *47*–*49*), while the ICG/Leci formula exhibited favorable kinetics and biocompatibility. The injection of ICG/Leci yielded less inflammatory infiltrate than either PBS or DFO, demonstrating its value in clinical translation.

However, there are still some issues that should be discussed. The first is the dose of ICG. On the one hand, setting wide applications in the clinic aside, ICG-related phototoxic effects still exist at high concentrations (*50*). However, the dose of ICG used in mice is 2.5 mg kg^−1^ and 0.5 mg kg^−1^ in human subjects, which is well below the previously reported safely injected dose of 5 mg kg^−1^ (*51*). Furthermore, ICG is almost exclusively transported by the liver (*52*, *53*) [volume: ~1.3 ml; weight of mouse: 20 g (*54*)]. Thus, it chelates 27 μM Fe^3+^ iron (*55*) (maximum in Hjv^−/−^) with 100 μM ICG. Thus, the 2.5 mg kg^−1^ dose of ICG could completely satisfy the reaction concentration of chelatable iron pool in mouse liver. Considering these two points, 2.5 mg kg^−1^ ICG satisfies security and validity in mice. More accurate calculations can be introduced to future dosage designs in mice or further pilot clinical studies. The second is ion selection. There are many kinds of metal ions (Zn^2+^, Cu^2+^, Mn^2+^, etc.) in the liver (*56*), and ICG may chelate with them as well (*57*, *58*). However, a higher abundance of iron (*59*) and a higher affinity between sulfonic acid structure–based ICG and Fe^3+^ finally contribute to high selectivity (*60*). Future experiments are needed to study more animal models and patients with iron-overload disorders to definitively determine the clinical benefit of ICG-based theranostic agents for LIC.

In summary, we repurposed ICG as a promising theranostic agent to specifically and sensitively diagnose and deplete deposited iron in the liver. This approach inhibits water exchange in the Fe^3+^ iron via a hydrophobic group of ICG, and the resulting quick *T*_1_SIR showed an excellent linear relationship, with LIC serving as a highly accessible iron quantitation method in vivo. The specific PA signal at 890 nm of ICG/Leci was markedly increased upon chelating with Fe^3+^, making it favorable for more sensitive iron detection to guide iron chelation therapy. The introduction of Leci also played an essential role in improving iron depletion efficacy and reducing iron toxicity. Our findings thus open up a personalized approach for iron-overloading patients to simultaneously gain better treatment planning and therapy.

## MATERIALS AND METHODS

### Materials

ICG was purchased from J&K Scientific Ltd. (Beijing, China). Lecithin was purchased from Sangon Biotech (Shanghai) Co. Ltd. MTT and 4′,6-diamidino-2-phenylindole (DAPI) were obtained from Sigma-Aldrich Co. Ltd. (MO, USA). Dulbecco’s modified Eagle’s medium and fetal bovine serum were purchased from Hyclone Inc. All other chemicals were purchased from Sigma-Aldrich in analytical grade and used without further purification.

### Computational methods

All calculations were carried out using Gaussian09. Optimization of closed shell singlet spin states was performed with restricted-B3LYP (RB3LYP), and the high spin states were performed using unrestricted-B3LYP (UB3LYP). A mixed basis set 6-31G(d) for the normal atoms and Stuttgart Dresden (SDD) for Fe were used for geometry optimizations and frequency analysis. Computed structures were illustrated using CYLView.

### Isothermal titration calorimetry

A MicroCal ITC 200 microcalorimeter (GE15 Healthcare) was used to measure the Fe^3+^ binding with ICG or ICG/Leci at 20°C. Hepes buffer (10 mM, pH 4.5), which mimicked acid environments in lysosomes, containing CH_3_OH, CHCl_3_, and *n*-hexane (15:6:1), was mixed and allowed to stand for 24 hours. ICG (2 mM), DFO (2 mM), and ICG/Leci (2 mM:4 mM) were injected 19 times (2 μl per time) into Fe^3+^ (0.1 mM). The data were repeated twice and analyzed by OriginPro 6s software.

### Optical activations of probes in solution

ICG, lecithin, and FeCl_3_ were dissolved in HCl (pH 4.5). The changes in fluorescence, absorbance, and optoacoustic signal of ICG and ICG/Leci upon addition of various concentrations of Fe^3+^ were recorded after different rounds of mixing. An ultraviolet-visible (UV-vis) system was applied to detect the absorbance changes from 200 to 1000 nm. Fluorescence production was recorded at an excitation wavelength of 680 nm. The photoacoustic signal was acquired at 890 nm (100% power, 40-dB gain, 21-MHz frequency).

### MRI experiment in solution

The MRI experiment was performed on a 1.5-, 3-, and 9.4-T MRI scanner. Different concentrations of Fe^3+^, Fe^3+^/Leci, Fe^3+^/ICG, Fe^3+^/ICG/Leci, ferritin, a-ferritin, ferritin/ICG, and a-ferritin/ICG at final Fe^3+^ concentrations (0, 0.0078, 0.0156, 0.0313, 0.0625, 0.125, and 0.25 mM) were dispersed in 2% agarose and recorded with an MRI scanner. The signal intensity was evaluated by analyzing the region of interest (ROI): ICG—0, 0.0156, 0.0313, 0.0625, 0.125, 0.25, and 0.5 mM; lecithin—0, 0.0313, 0.0625, 0.125, 0.25, 0.5, and 1 mM.

### MRI experiment in liver tissues

Animal experiments were approved by the Animal Care and Use Committee of Xiamen University. WT 129S1/SvTmJSlac and c57 mice were obtained from Beijing Vital River Laboratory Animal Technology Co. Ltd. The isolation of liver tissue was performed in 129S1/SvTmJSlac mice after being anesthetized with 1% isoflurane. Liver tissues were subsequently collected from the abdomen and cut into small pieces and then injected with ICG (10 μl, 100 μM), Fe^3+^ (10 μl, 25 μM), or Fe^3+^ (5 μl, 50 μM) following ICG (5 μl, 200 μM). The MRI experiment was performed under a 9.4-T MRI scanner with the same parameters as above.

### Cytotoxicity test

Primary hepatocytes were harvested from the livers of mice and seeded into six-well plates at a density of 5 × 10^5^ cells per well at 37°C under 5% CO_2_ before they were incubated with Fe^3+^ at final Fe^3+^ concentrations of 0, 5, 10, 20, 50, 100, 200, and 500 μM at 37°C for 4 hours in a CO_2_ incubator. The cells were then incubated with ICG/Leci (ICG, 40 μM; Leci, 80 μM) for another 20 hours. Next, the medium was replaced with culture medium, and 10 μl of MTT solution (5 mg ml^−1^, dissolved in PBS) was added to each well. The mixture was then incubated for 4 hours. The MTT solution was carefully removed, and 150 μl of dimethyl sulfoxide was added for 10 min to solubilize the violet formazan crystals. Thereafter, the absorbance of each well was measured using a Thermo Fisher Scientific Multiskan MK3 ELISA reader (Thermo Fisher Scientific) at 570 nm. As for CCK8 assays, 10 μl of CCK solution was added to each well at the end of 20-hour incubation time and further incubated for another 2 hours, and the absorbance was recorded at 450 nm.

### MRI, fluorescence, and PAI of cells

Various concentrations of Fe^3+^ were added to primary hepatocytes harvested as above. These cells were allowed to adhere to glass culture dishes. Four hours later, ICG/Leci (ICG, 40 μM; lecithin, 80 μM) was added and incubated for another 2 hours. Next, DAPI was incubated with cells for 15 min (room temperature). The dishes were washed with PBS three times and prepared for fluorescence imaging. Similarly, 1 × 10^7^ cells cultured with Fe^3+^ and ICG/Leci were detached from dishes, washed three times, and fixed into a cell ball with 1% paraformaldehyde for 1 hour. The cell balls were then subjected to MRI, fluorescence, and PAI.

### Western blot analysis

Mouse primary hepatocytes cultured with Fe^3+^ and ICG/Leci were lysed using radioimmunoprecipitation assay lysis buffer, and the total protein was loaded on a 12% SDS–polyacrylamide gel electrophoresis. The samples were then transferred to nitrocellulose membranes. After blocking with 5% bovine serum albumin, the material was incubated overnight with a rabbit anti-human H-ferritin primary antibody at 1:1000 dilution (Abcam, ab65080), rabbit anti-mouse glyceraldehyde-3-phosphate dehydrogenase (GAPDH) at 1:10,000 dilution (ABclonal, AC001), rabbit anti-mouse TfR1 at 1:1000 dilution (Abcam, ab65080), rabbit anti-human ferroportin at 1:1000 dilution (Alpha Diagnostics, MTP11-A), and anti-rabbit immunoglobulin G secondary antibodies at 1:2500 dilution (ABclonal, AS014); the samples were detected with the ECL System (Pierce).

### In vivo biodistribution and MRI/PAI

The Hfe^−/−^, Hjv^−/−^, and HBV-Tg mice were maintained in the animal facility of State Key Laboratory of Molecular Vaccinology and Molecular Diagnostics with a 12-hour light/12-hour dark cycle at ~18° to 23°C and ~50% humidity. All animals received humane care, and all experiments were conducted in compliance with institutional and national guidelines and supervised by staff from the Xiamen University. Mice (8 weeks old) received ICG and ICG/Leci (ICG dose: 2.5 mg kg^−1^) via tail vein and were anesthetized with 1% isoflurane. Before the MRI/PAI, real-time fluorescence imaging (Carestream FX Pro) was used to curve the biodistribution of ICG and ICG/Leci in livers at 672 nm.

MRI used a 9.4-T magnetic field micro-MR scanner (Bruker) to record *T*_1_-weighted MR images from the abdomen with the following parameters: repetition time (TR), 1000 ms; echo time (TE), 8.5 ms; flip angle, 180°; number of excitations, 4; matrix size, 256 × 256; field of view (FOV), 4 × 4 cm^2^; slice number, 13; and slice thickness, 1 mm. For PAI, a Vevo LAZR system with consistent parameters (100% power; frequency at 21 MHz; gain at 40 dB; excitation at 890 nm) was also conducted after intravenous injection of ICG or ICG/Leci (ICG dose: 2.5 mg/kg). Imaging was done at *t* = 0, 1, 4, 8, 12, and 24 hours. Signal intensities of both images were measured in a defined ROI, and the signal-to-noise ratio values were calculated using different formulas.

### MRI techniques and image analysis of subjects before and after ICG injection

Three subjects (male) were recruited. After having blood drawn to detect liver function, serum ferritin, and transferrin saturation, the subjects received an iodine/ICG allergy test and were treated with ICG (0.5 mg kg^−1^) via cubital vein injection. MRI measurements were conducted using an 18-element Tim coil at a field strength of 3.0 T (MAGNETOM Skyra, Siemens Medical, USA). Liver *T*_1_ MRI measurement was conducted using *T*_1_-weighted VIBE (Volume Interpolated Breath-Hold Examination) sequences with the following parameters: TR, 3.97 ms; TE, 1.29 ms; and FOV, 308 × 380 cm^2^. The muscle-to-liver *T*_1_ ratio was calculated in eight ROIs of two slices per time point. The eight ROIs were placed in the eight segments of the liver. The study was approved by the Institutional Review Board (IRB) of Xiang’an Hospital of Xiamen University (IRB no. XAHLL2020004) and registered at ClinicalTrials.gov (ID: ChiCTR2000035648). The subjects signed and gave written informed consent.

### Clinical liver tissue specimen collection and analysis of Prussian blue images and PA images

Liver tissue specimens from the biopsy with pathologically proven iron overload and corresponding normal tissue samples were collected at Mengchao Hepatobiliary Hospital. The acquisition of samples was approved by the ethics committees of Mengchao Hepatobiliary Hospital, and each patient gave written informed consent. The 6-mm sections were stained with Prussian blue and ICG/Leci after being dewaxed. These clinical specimens were further imaged by PAI. In addition, the gray scale of Prussian blue staining images and photoacoustic images was analyzed by ImageJ software to determine the signal distribution.

### Iron excretion efficacy and elimination studies in vivo

PBS (250 μl), ICG (2.5 mg kg^−1^), and ICG/Leci (ICG, 2.5 mg kg^−1^) were tail vein–injected into 8-week-old Hfe^−/−^ mice (*n* = 5 per group) with a single dose. Another five mice were intravenously treated with equivalent doses of DFO as a positive control group. After the injection, these mice were put into metabolic cages with a regular diet. Excreted urine and feces were collected at different time points (*t* = 1, 3, 6, 9, 12, 24, and 36 hours) and digested by a mixture of HNO_3_ and H_2_O_2_ (1:3). The iron concentration of these samples was detected by ICP-MS to calculate the urinary and feces iron excretion.

The iron elimination studies in the organs of the above group of mice were examined following an intravenous injection with the same drugs once every 2 days. After 2 weeks, mice were euthanized with 1% isoflurane, and the blood was collected from the eye socket.

The serum ferritin efficacy study for these blood samples was measured by ELISA conducted with an automatic biochemical analyzer (BS-220, Mindray). Organs (liver, spleen, kidneys, heart, lungs, and brain) were then harvested from sacrificed mice. The weighed part of the liver and spleen was also digested with a mixture of HNO_3_ and H_2_O_2_ (1:3). ICP-MS was used to detect the iron concentration. Part of the liver was fixed by paraformaldehyde and stained with Prussian blue. The remaining organs, including the liver, spleen, kidneys, heart, lungs, and brain, were fixed and stained with H&E.

## Notes

View/request a protocol for this paper from *Bio-protocol*.
