# Supplementary material for: Repurposing ICG enables MR/PA imaging signal amplification and iron depletion for iron-overload disorders
Source: Sci Adv. 2021 Dec 17;7(51):eabl5862. doi: 10.1126/sciadv.abl5862 (PMC8682994; doi:10.1126/sciadv.abl5862)
Supplement: Supplementary file 1 — Supplementary Text Figs. S1 to S22 [file sciadv.abl5862_sm.pdf]

Science Advances
AAAS

Supplementary Materials for

Repurposing ICG enables MR/PA imaging signal amplification and iron depletion for iron-overload disorders

Huirong Lin, Yu Zhou, Jiaming Wang, Huimeng Wang, Tianhong Yao, Hu Chen, Huili Zheng, Yang Zhang, En Ren, Lai Jiang, Chengchao Chu, Xiaoyuan Chen, Jingsong Mao*, Fudi Wang*, Gang Liu*

*Corresponding author. Email: gangliu.cmitm@xmu.edu.cn (G.L.); fwang@zju.edu.cn (F.W.); maojingsong163@163.com (J.M.)

Published 17 December 2021, *Sci. Adv.* **7**, eabl5862 (2021)
DOI: 10.1126/sciadv.abl5862

This PDF file includes:

Supplementary Text
Figs. S1 to S22

Supplementary Text

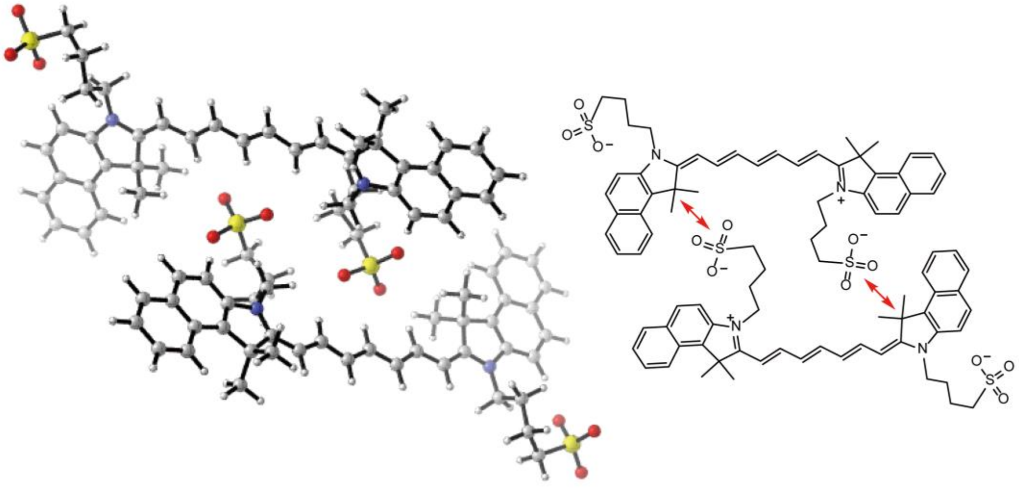

ICG-ICG $\Delta E = -20.2$ kcal mol^{-1}

Figure S1. The aggregation modes between ICG molecules. All calculations were carried out using Gaussian09. The optimization of closed shell singlet spin states was performed with restricted-B3LYP (RB3LYP), and the high spin states were performed using unrestricted-B3LYP(UB3LYP). Computed structures are illustrated using CYLView. ICG molecules can aggregate mainly through the electrostatic interaction between the sulfonate anion of one ICG molecule and the quaternary ammonium of the other ICG molecule. The interaction energy between ICG molecules is -20.2 kcal mol^{-1}, which indicates that ICG molecules could self-polymerize slowly and requires high concentration.

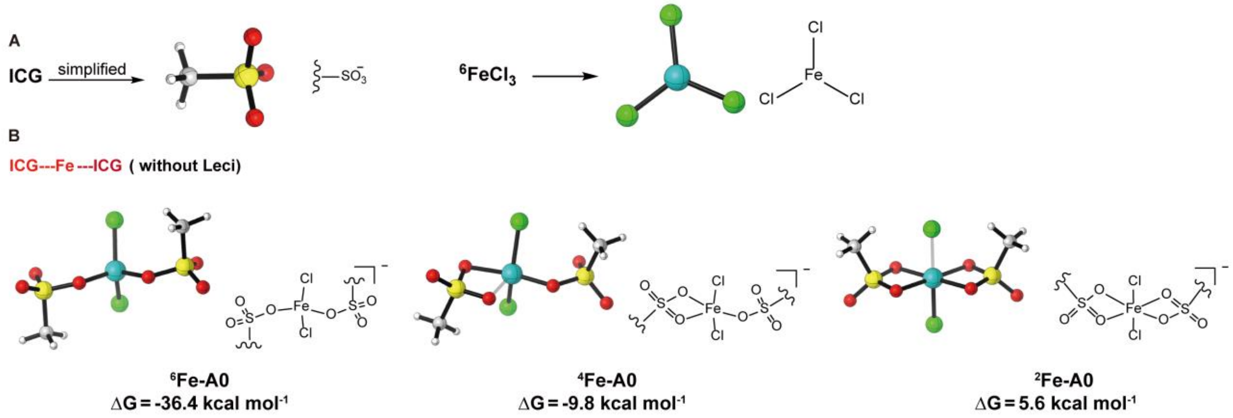

Figure S2. Aggregation modes between Fe^{3+} and ICG. A mixed basis set 6-31G(d) for the normal atoms and SDD for Fe were employed for geometry optimizations and frequency analysis. Computed structures are illustrated using CYLView. When ferric chloride is added to ICG, several aggregation modes are investigated with DFT calculation. In each aggregation mode, a chlorine atom of $FeCl_3$ leaves from Fe center to vacate the coordination site for ICG. On the other hand, iron is one of the first-row TMs, which are more likely to involve open-shell electronic structures and are often associated with multiple spin states close in energy; in this work multi-state structures are considered for each species involving iron. However, the calculation of high spin states of the aggregation system is very time-consuming. The simplified models for ICG are adopted for DFT calculation to save computation time. In the simplified model of ICG, the sulfonate is kept. Here, we use superscripts 2/4/6 on the upper-left of a given complex (e.g., 2/4/6Fe-A0) to denote the doublet/quartet/sextet state. **A,** simplified models of ICG in the DFT calculation. **B,** the aggregation complexes between Fe^{3+} and ICG.

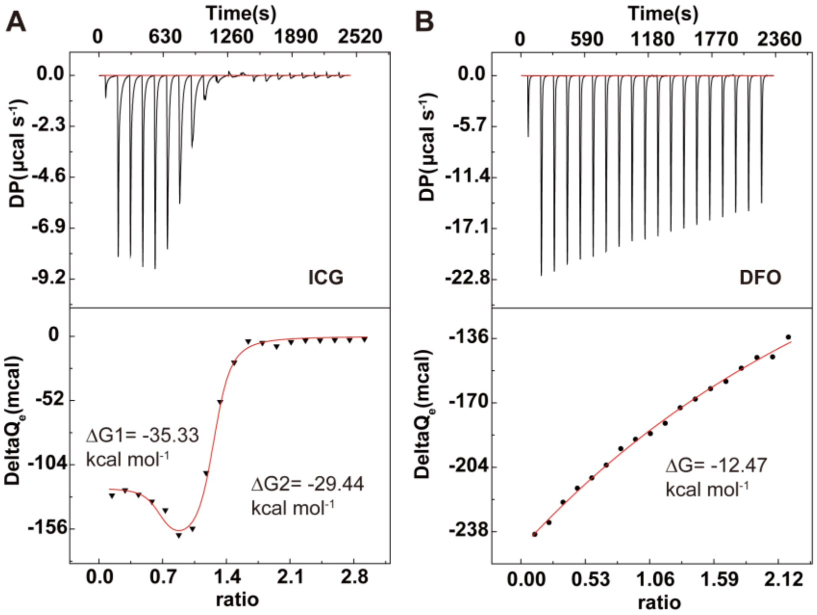

Figure S3. ITC data for the binding of ICG or DFO with Fe^{3+}. ITC data for the binding of ICG (2 mM) **(A)**, DFO (2 mM) **(B)** onto Fe^{3+} (0.1 mM) in HEPES buffer at pH 4.5, which mimicked acid environments in lysosomes.

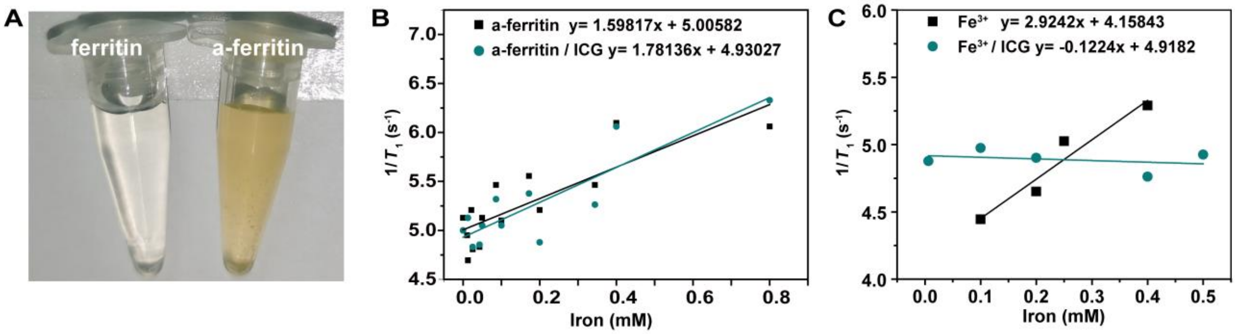

Figure S4. Photos and r_1 of ferritin and aggregated ferritin. **A,** photos of ferritin and aggregated ferritin (a-ferritin) (Iron, 1 mM). Ferritin was expressed in Escherichia coli. After purification, the proteins were biomineralized to prepare the a-ferritin (yellow). **B,** the correlation between T_1 and iron concentration of a-ferritin and a-ferritin/ICG was measured in 9.4T of a magnetic field at pH 6.8, and correlation between T_1 and iron concentration of Fe^{3+} or Fe^{3+}/ICG under the same solution condition (**C**).

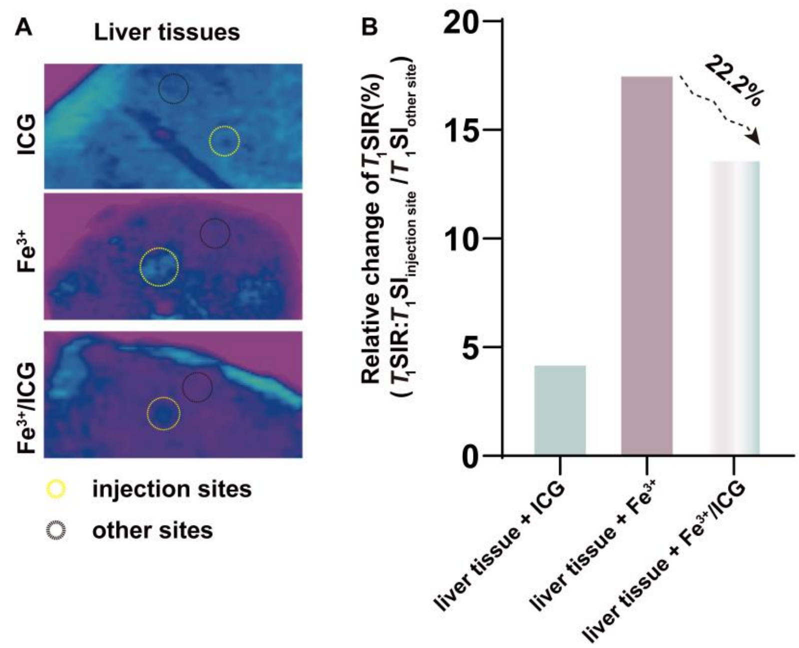

Figure S5. Relative change of T_1SIR in liver tissues *in vitro*. A, representative T_1 signal images were obtained in a 9.4 T MRI system. **B,** the relative change of T_1SIR in liver tissues *in vitro* with ICG (10 μL, 100 μM), Fe^{3+} (10 μL, 25 μM) or Fe^{3+} (5 μL, 50 μM) following ICG treatment (5 μL, 200 μM).

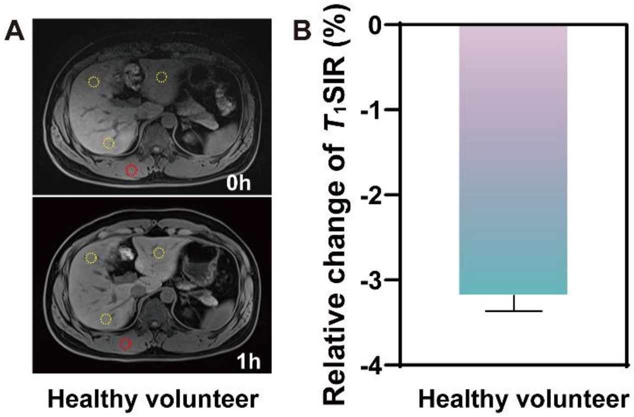

Figure S6. Relative change of T_1SIR in liver tissue of healthy volunteer. A, a healthy subject (male) was treated with ICG (0.5 mg kg^{-1}) by Cubital vein injection. T_1SI of the liver was measured in 8 ROIs of 2 slices per time point. 8 ROIs were placed in the 8 segments of the liver, and the relative change of T_1SIR =100* $[(T_1SI_M / T_1SI_L)^{0h}$ **-** $(T_1SI_M / T_1SI_L)^{nh}]/ (T_1SI_M / T_1SI_L)^{0h}$ was calculated **(B).** (mean ± s.d., n = 8)

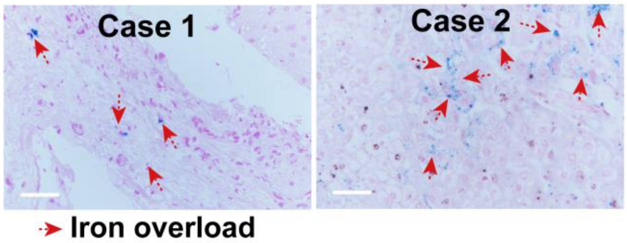

Figure S7. Representative Prussian blue-stained liver sections of patients with chronic viral hepatitis-related hepatocellular carcinoma. Case 1: LIC = 463.89 ± 10.03 μg g^{-1} (mild positive Prussian blue staining)； Case 2: LIC = 677.54 ± 146.31 μg g^{-1} (moderate positive Prussian blue staining). Scale bar, 200 μm.

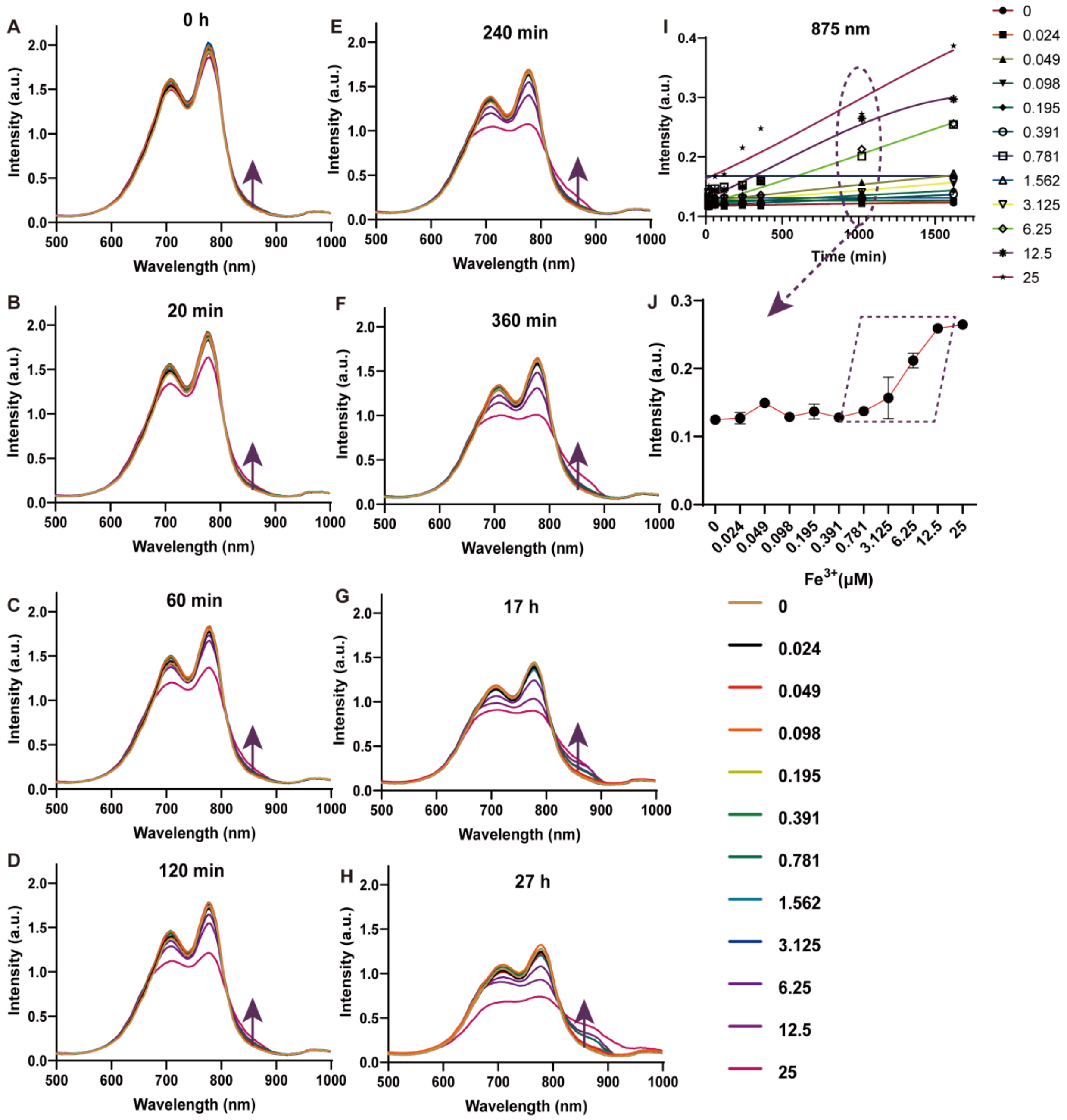

Figure S8. Photophysical characterization of ICG in the presence of different Fe^{3+} concentrations. (**A-H**) UV-vis absorption spectra of ICG (100 μM) upon incubation with varied concentrations of Fe^{3+} (0-25 μM) at pH 4.5 at different time points. (**I-J**) Quantitative 875 nm absorbance of Fe^{3+}/ ICG at pH 4.5. (mean ± s.d., n = 3)

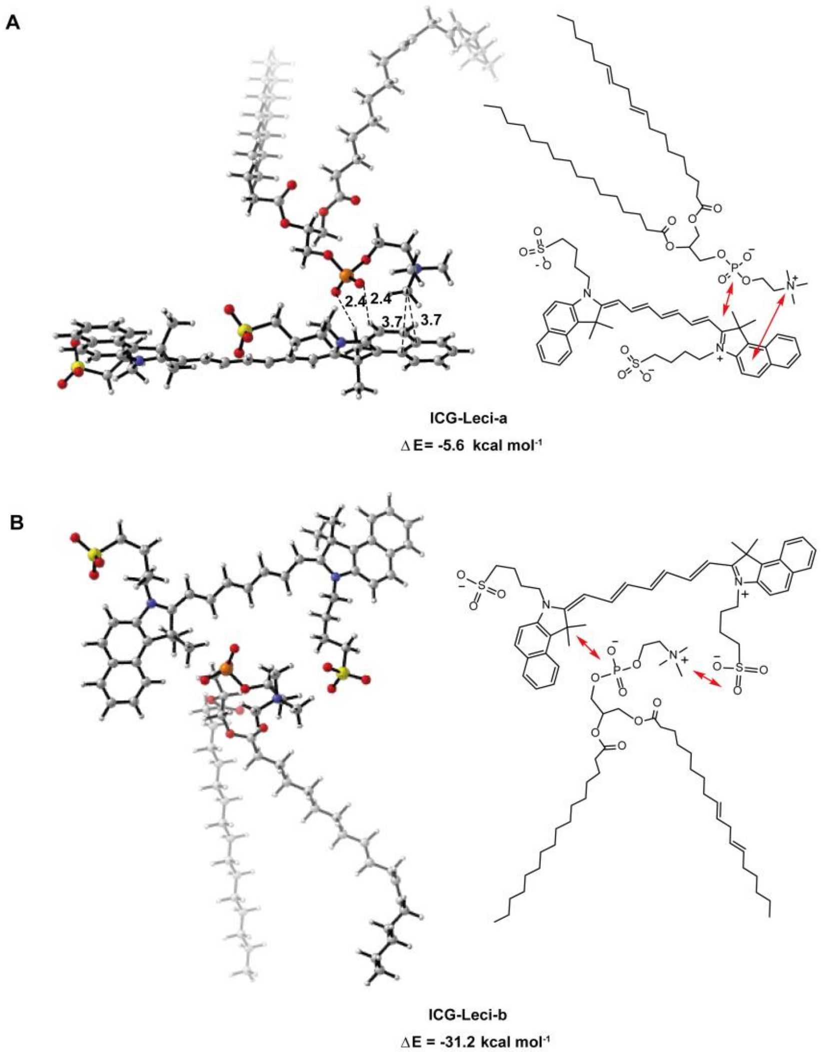

Figure S9. Aggregation modes between ICG and Leci. A, in one mode**,** the aggregation is mainly attributed to two interactions: (i) the cation-π interaction between the choline portion of leci and the aromatic ring of ICG and (ii) the electrostatic interaction between the phosphate anion of Leci and the quaternary ammonium of ICG. **B,** in the other mode, the aggregation should be formed due to two electrostatic interactions: (i) the electrostatic interaction between the choline portion of Leci and sulfonate group of ICG and (ii) the electrostatic interaction between the phosphate anion of Leci and the quaternary ammonium of ICG. The interaction energies between ICG and Leci in the two modes are -5.6 kcal mol^{-1} and -31.2 kcal mol^{-1}, respectively. It is believed that the second mode should be more favorable.

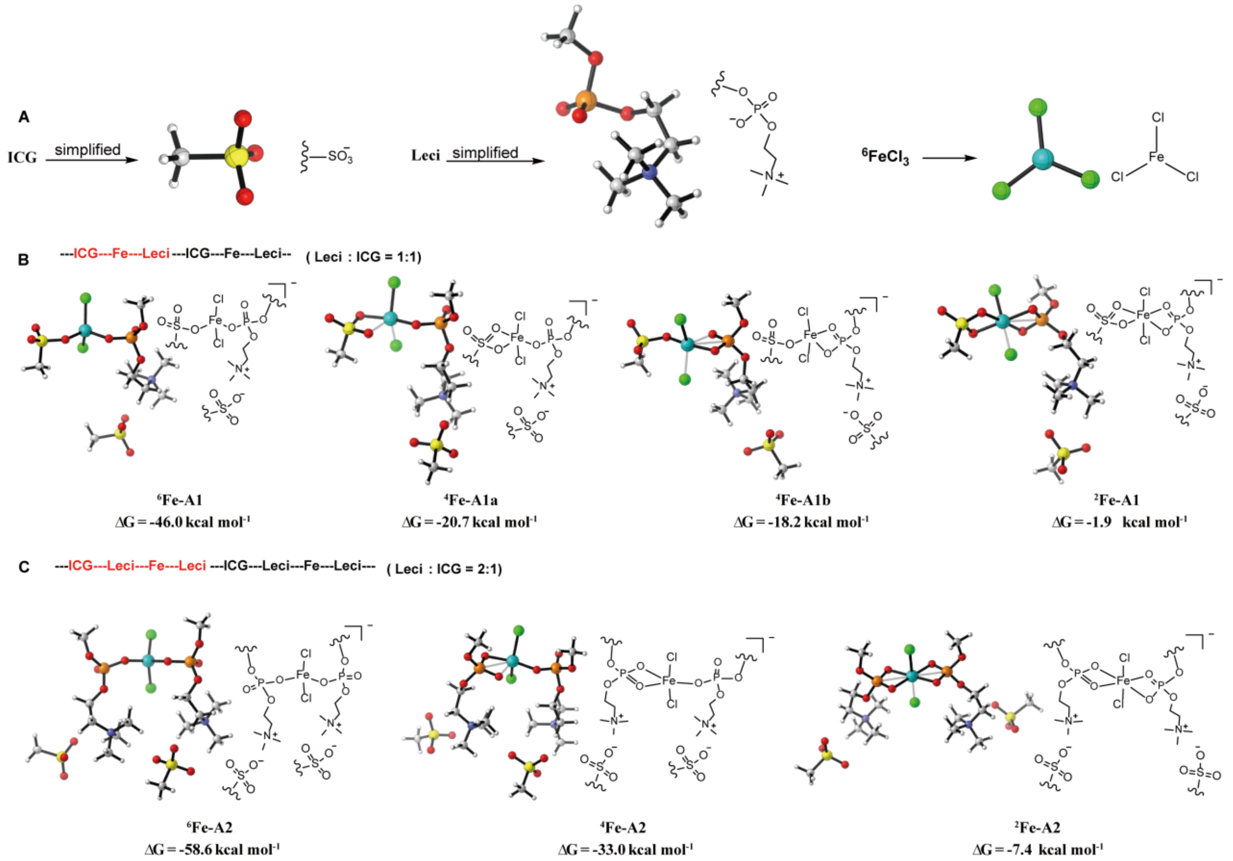

Figure S10. Aggregation modes among Fe^{3+}, ICG and ICG/Leci. We use superscripts 2/4/6 on the upper-left of a given complex (e.g., 2/4/6Fe-A0) to denote the doublet/quartet/sextet state. **A,** simplified models of ICG and Leci in the DFT calculation. **B,** the aggregation complexes between iron, Leci, and ICG when Leci: ICG = 2: 1. **C,** the aggregation complexes between iron, Leci, and ICG when Leci: ICG = 2: 1.

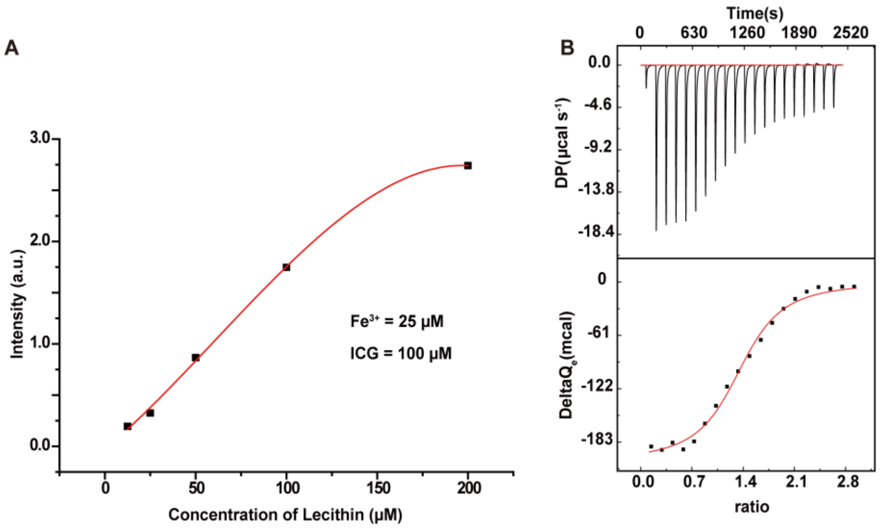

Figure S11. UV-vis absorption and ITC data for the binding of ICG/Leci onto Fe^{3+}. A, UV-vis absorption spectra at 890 nm of Fe^{3+}(25 μM)/ICG (100 μM) upon incubation with different concentrations of Lecithin (0-200 μM) at pH 4.5 in 4h. **B,** ITC data for the binding of ICG (2 mM)/Leci (4 mM) onto Fe^{3+} (0.1 mM) in HEPES buffer at pH 4.5, which mimicked acid environments in lysosomes.

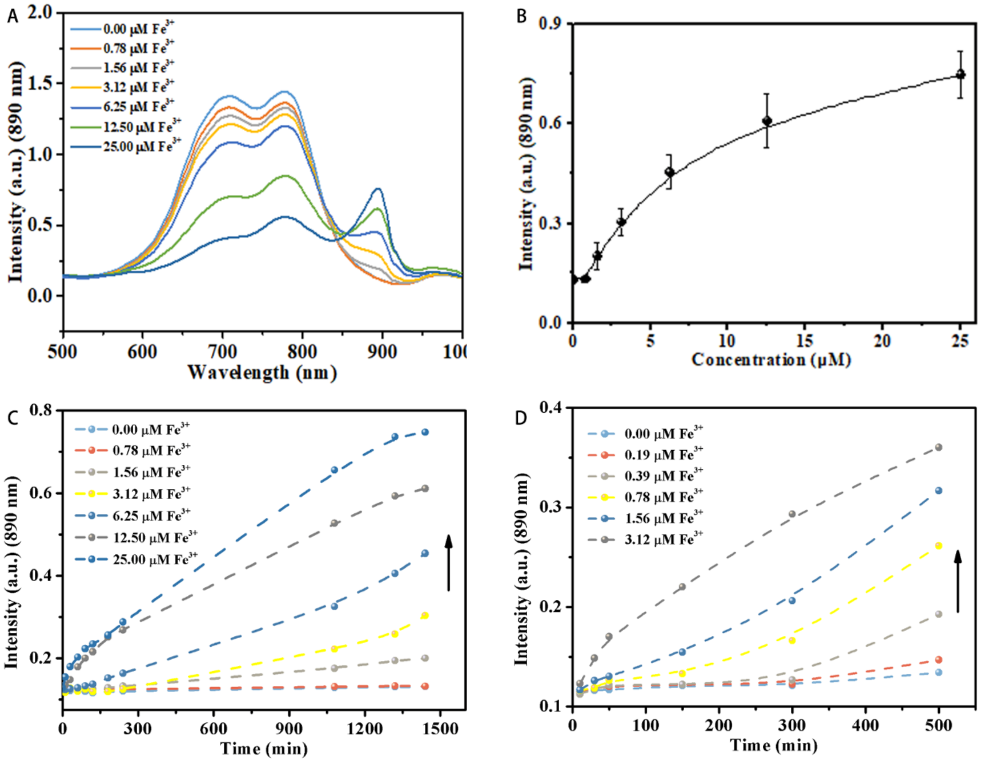

Figure S12. UV-vis absorption and its change for the binding of ICG/Leci onto Fe^{3+}. A, UV-vis absorption spectra of ICG (100 µM) /Leci (200 µM) upon incubation with different concentrations of Fe^{3+} (0-25 µM) at pH 7.4 and the plots of absorbance at 890 nm vs. biomarker concentration. **B,** Quantitative 890 nm absorbance of Fe^{3+}/ ICG/Leci at pH 7.4 **(C)** and pH 4.5 **(D)**.

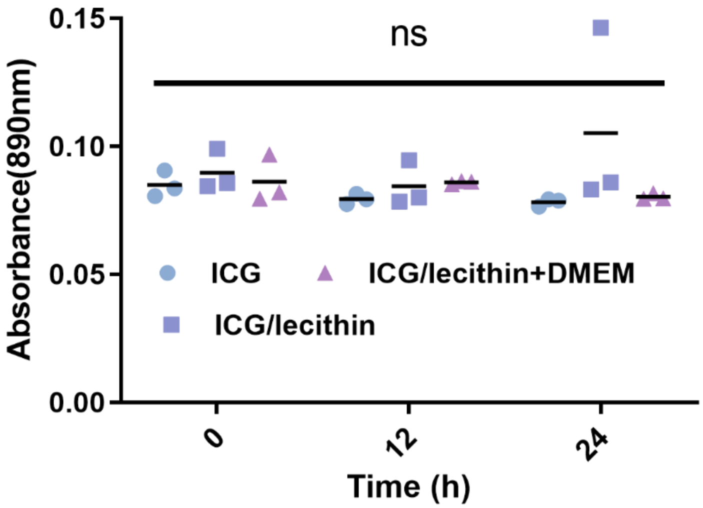

Figure S13. UV-vis absorbance at 890 nm of ICG, ICG/Leci, ICG/Leci without adding Fe^{3+}. Quantitative 890 nm absorbance of ICG, ICG/Leci, ICG/Leci + DMEM at pH 4.5 in 0, 12, and 24 h. (ns, not significant by unpaired t-test (two-tailed))

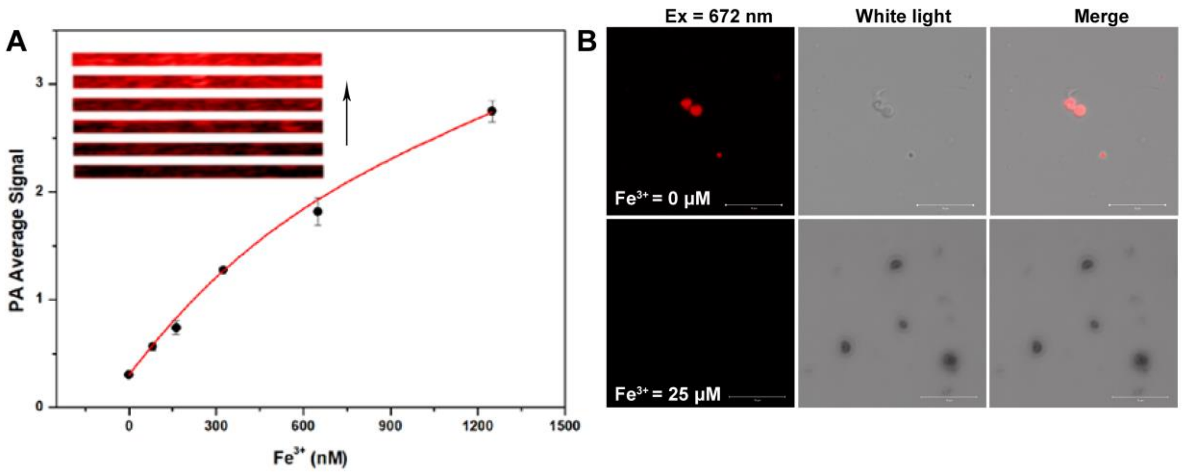

Figure S14. Photoacoustic/ fluorescence response of the probes to Fe^{3+}. A, Photoacoustic response of the probes to Fe^{3+} (0-25 μM) at pH 7.4. **B,** the Fe^{3+}/ICG/Leci confocal fluorescence images before and after adding Fe^{3+}, indicating the complex interactions between Fe^{3+} and drug ligands (Ex, 672 nm).

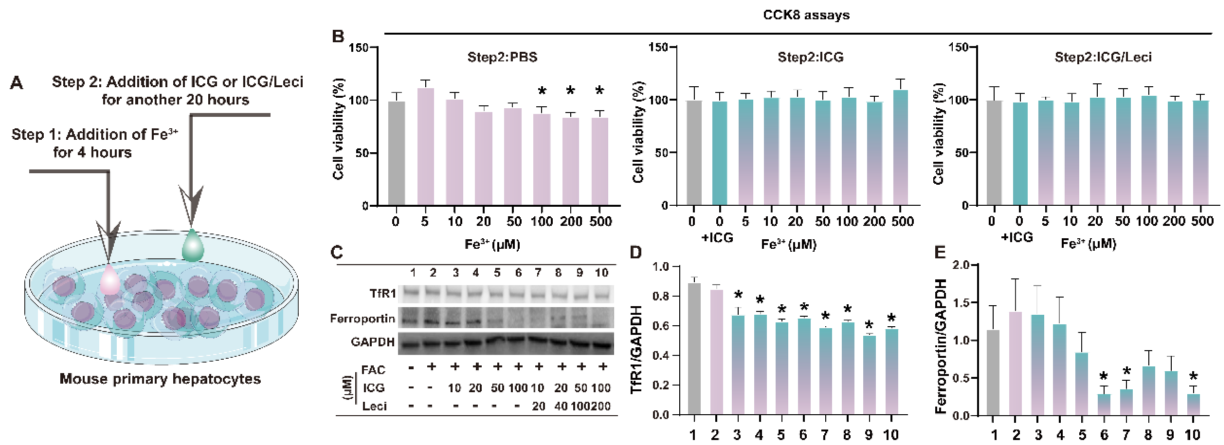

Figure S15. CCK8 assays and cell signaling regulation. A, design of cell experiments. The viability of mouse primary hepatocytes was assessed by CCK8 assays after adding Fe^{3+} followed by treatment with PBS **(B)**, free ICG **(C)** and ICG/Leci **(D)**, and western blots were carried out for protein detection **(C)**. The amount of FfR1 protein **(D)** and Ferroportin protein **(E)** were quantified, normalized to the amount of GAPDH. The Fe^{3+}/ICG/Leci shows no obvious cytotoxicity on mouse primary hepatocytes with significantly reduced levels of ferroportin protein and TfR1 protein. (mean ±s.d., n = 4/group, *$p < 0.05$ by unpaired t-test (two-tailed)).

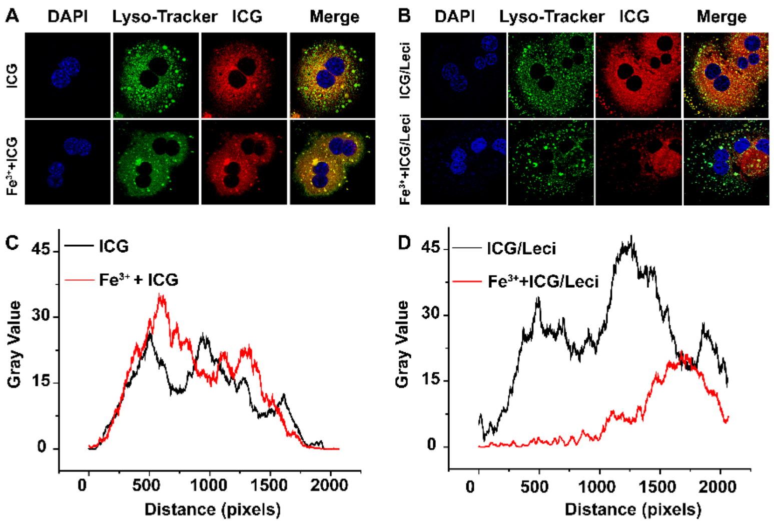

Figure S16. CLSM images of mouse primary hepatocytes cells after being treated with different agents. CLSM images of cells after being incubated with free ICG, Fe^{3+} following ICG **(A)**, ICG/Leci and Fe^{3+} following ICG/Leci **(B)**. And the image pixels were respectively recorded by Image J software **(C, D)**.

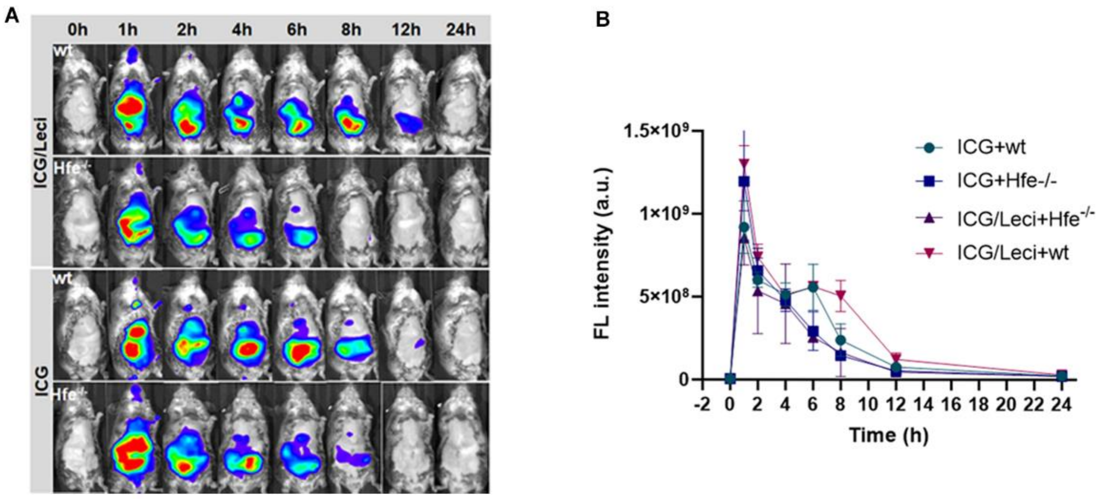

Figure S17. Biodistribution of the ICG and ICG/Leci *in vivo*. Representative fluorescence images **(A)** and the corresponding quantitative measurements **(B)** of wt or Hfe$^{-/-}$ mice with i.v. injection of ICG or ICG/Leci for 0, 1, 2, 4, 6, 8, 12, and 24 h. (mean ± s.d., n = 3).

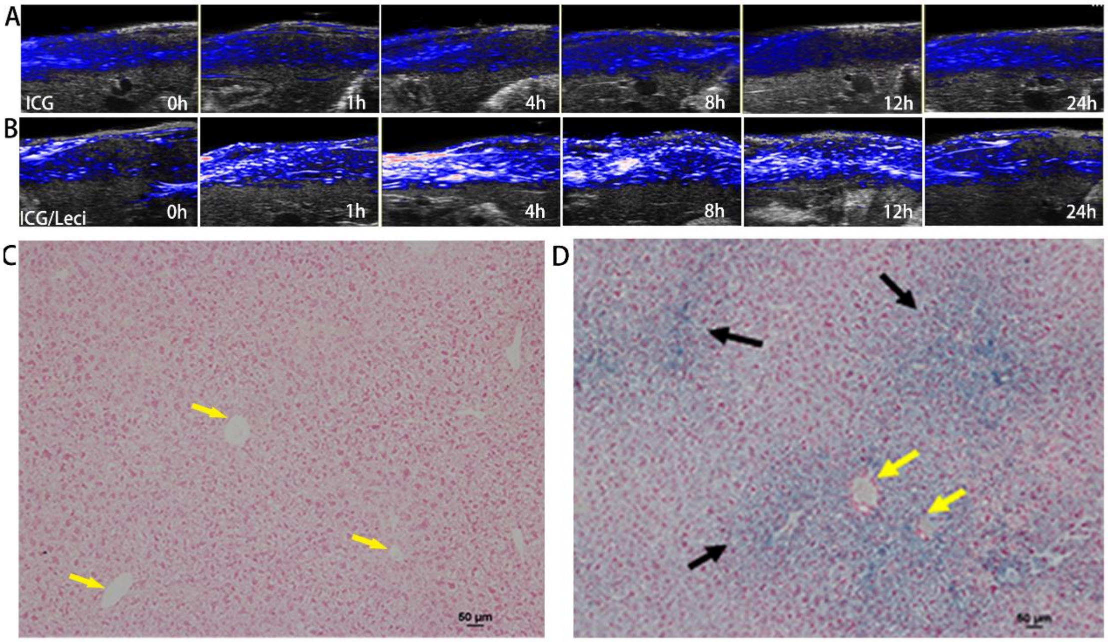

Figure S18. PA imaging and Prussian blue-staining. Representative PA images *in vivo* at 890 nm of Hjv$^{-/-}$ mice following intravenous injection of free ICG **(A)** and ICG/Leci **(B)**. Prussian blue staining of representative liver sections of wt **(C)** and Hjv$^{-/-}$ mice **(D)**. The yellow arrows point to hepatic veins and the black arrows point to significant iron deposition around the veins.

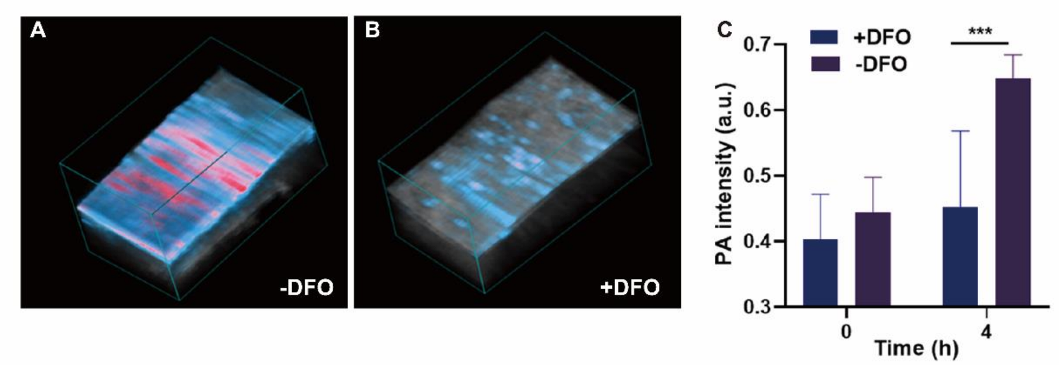

Figure S19. PA imaging to monitor iron depletion after DFO treatment. Representative 3D PA images **(A, B)** and signal intensity in 0 h and 4 h **(C)** at 890 nm of non-DFO-treated and DFO-treated $Hfe^{-/-}$ mice following intravenous injection of ICG/Leci (ICG dose: 2.5 mg kg^{-1}). (mean ± s.d., n = 5/group, ***$p < 0.001$ by unpaired t-test (two-tailed)).

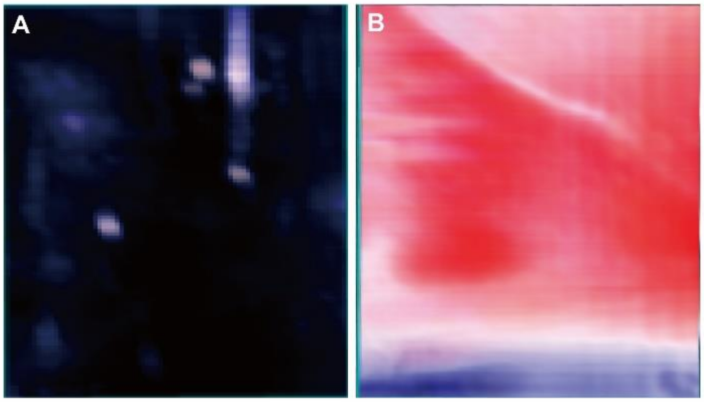

Figure S20. PA imaging of iron deposited liver slices with ICG staining. Without **(A)** and with **(B)** ICG staining following PA imaging of liver slice from the iron overload patients confirmed by liver biopsy measurements.

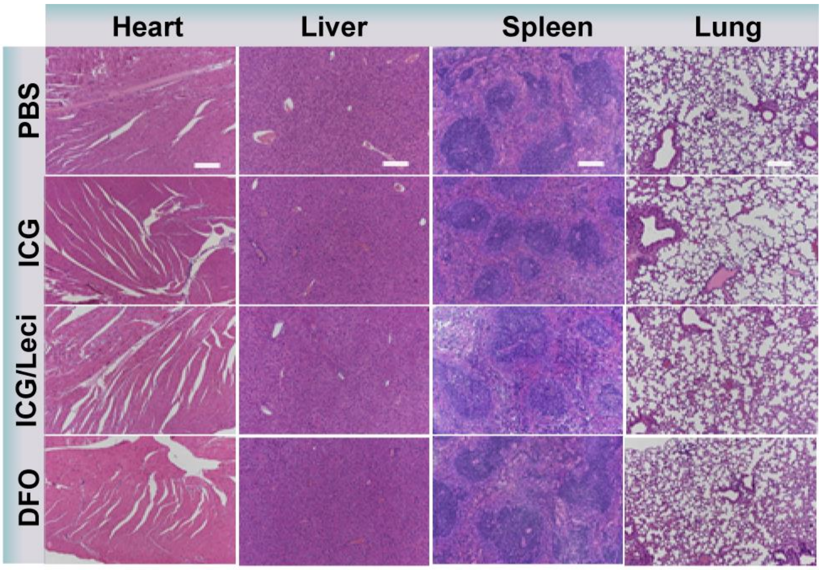

Figure S21. H&E-stained sections of the heart, liver, spleen, and lung. Scale bar: 100 μm.

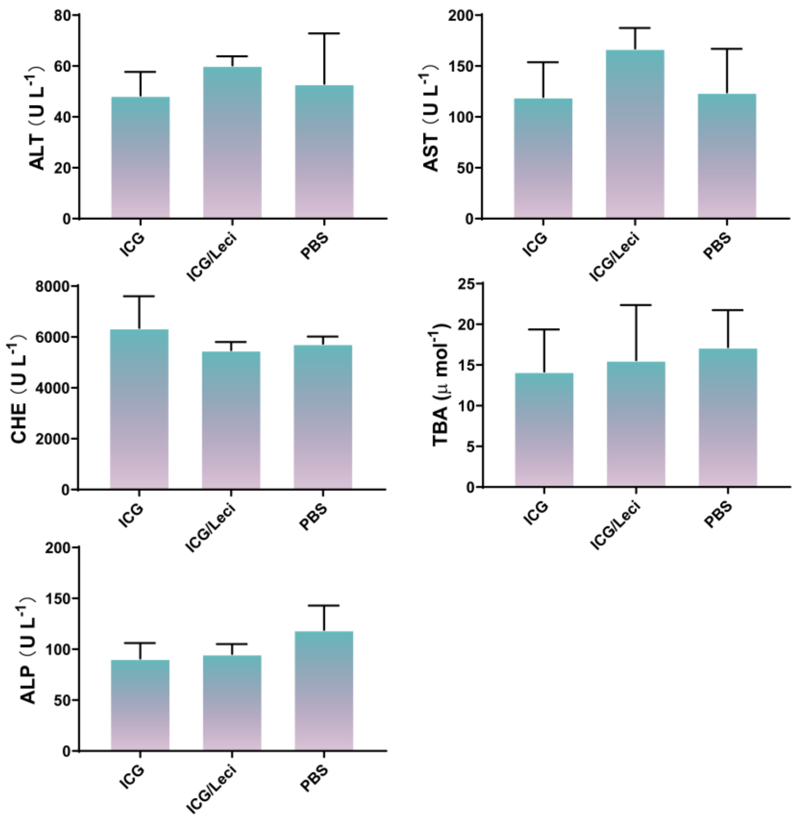

Figure S22. Blood biochemical assays. Blood biochemical assays using ELISA kits for cholinesterase (CHE), total bile acid (TBA), alkaline phosphatase (ALP) aspartate aminotransferase (AST), alanine aminotransferase (ALT) for female c57 mice (8-week-old) after intravenous injection of ICG, ICG/Leci, PBS (ICG does, 2.5 mg kg^{-1}) every other day for 7 times. (mean ± s.d., n = 3)
